# Growth-regulating factor 15-mediated gene regulatory network enhances salt tolerance in poplar

**DOI:** 10.1093/plphys/kiac600

**Published:** 2022-12-26

**Authors:** Weijie Xu, Yue Wang, Jianbo Xie, Shuxian Tan, Haofei Wang, Yiyang Zhao, Qing Liu, Yousry A El-Kassaby, Deqiang Zhang

**Affiliations:** National Engineering Laboratory for Tree Breeding, College of Biological Sciences and Technology, Beijing Forestry University, No. 35, Qinghua East Road, Beijing 100083, P. R. China; Key Laboratory of Genetics and Breeding in Forest Trees and Ornamental Plants, Ministry of Education, College of Biological Sciences and Technology, Beijing Forestry University, No. 35, Qinghua East Road, Beijing 100083, P. R. China; National Engineering Laboratory for Tree Breeding, College of Biological Sciences and Technology, Beijing Forestry University, No. 35, Qinghua East Road, Beijing 100083, P. R. China; Key Laboratory of Genetics and Breeding in Forest Trees and Ornamental Plants, Ministry of Education, College of Biological Sciences and Technology, Beijing Forestry University, No. 35, Qinghua East Road, Beijing 100083, P. R. China; National Engineering Laboratory for Tree Breeding, College of Biological Sciences and Technology, Beijing Forestry University, No. 35, Qinghua East Road, Beijing 100083, P. R. China; Key Laboratory of Genetics and Breeding in Forest Trees and Ornamental Plants, Ministry of Education, College of Biological Sciences and Technology, Beijing Forestry University, No. 35, Qinghua East Road, Beijing 100083, P. R. China; National Engineering Laboratory for Tree Breeding, College of Biological Sciences and Technology, Beijing Forestry University, No. 35, Qinghua East Road, Beijing 100083, P. R. China; Key Laboratory of Genetics and Breeding in Forest Trees and Ornamental Plants, Ministry of Education, College of Biological Sciences and Technology, Beijing Forestry University, No. 35, Qinghua East Road, Beijing 100083, P. R. China; National Engineering Laboratory for Tree Breeding, College of Biological Sciences and Technology, Beijing Forestry University, No. 35, Qinghua East Road, Beijing 100083, P. R. China; Key Laboratory of Genetics and Breeding in Forest Trees and Ornamental Plants, Ministry of Education, College of Biological Sciences and Technology, Beijing Forestry University, No. 35, Qinghua East Road, Beijing 100083, P. R. China; National Engineering Laboratory for Tree Breeding, College of Biological Sciences and Technology, Beijing Forestry University, No. 35, Qinghua East Road, Beijing 100083, P. R. China; Key Laboratory of Genetics and Breeding in Forest Trees and Ornamental Plants, Ministry of Education, College of Biological Sciences and Technology, Beijing Forestry University, No. 35, Qinghua East Road, Beijing 100083, P. R. China; CSIRO Agriculture and Food, Black Mountain, Canberra, ACT 2601, Australia; Department of Forest and Conservation Sciences, Faculty of Forestry, Forest Sciences Centre, University of British Columbia, Vancouver, BC V6T 1Z4, Canada; National Engineering Laboratory for Tree Breeding, College of Biological Sciences and Technology, Beijing Forestry University, No. 35, Qinghua East Road, Beijing 100083, P. R. China; Key Laboratory of Genetics and Breeding in Forest Trees and Ornamental Plants, Ministry of Education, College of Biological Sciences and Technology, Beijing Forestry University, No. 35, Qinghua East Road, Beijing 100083, P. R. China

## Abstract

Soil salinity is an important determinant of crop productivity and triggers salt stress response pathways in plants. The salt stress response is controlled by transcriptional regulatory networks that maintain regulatory homeostasis through combinations of transcription factor (TF)–DNA and TF–TF interactions. We investigated the transcriptome of poplar 84 K (*Populus alba* × *Populus glandulosa*) under salt stress using samples collected at 4- or 6-h intervals within 2 days of salt stress treatment. We detected 24,973 differentially expressed genes, including 2,231 TFs that might be responsive to salt stress. To explore these interactions and targets of TFs in perennial woody plants, we combined gene regulatory networks, DNA affinity purification sequencing, yeast two-hybrid-sequencing, and multi-gene association approaches. Growth-regulating factor 15 (PagGRF15) and its target, high-affinity K^+^ transporter 6 (*PagHAK6*), were identified as an important regulatory module in the salt stress response. Overexpression of *PagGRF15* and *PagHAK6* in transgenic lines improved salt tolerance by enhancing Na^+^ transport and modulating H_2_O_2_ accumulation in poplar. Yeast two-hybrid assays identified more than 420 *PagGRF15*-interacting proteins, including ETHYLENE RESPONSE FACTOR TFs and a zinc finger protein (C2H2) that are produced in response to a variety of phytohormones and environmental signals and are likely involved in abiotic stress. Therefore, our findings demonstrate that PagGRF15 is a multifunctional TF involved in growth, development, and salt stress tolerance, highlighting the capability of a multifaceted approach in identifying regulatory nodes in plants.

## Teaser

Growth-regulating factor 15 in poplar acts as a multifunctional transcription factor involved in growth, development, and salt stress tolerance through a multi-level regulation network.

## Introduction

Soil salinity is a major abiotic constraint on crop productivity (Pitman et al., 2002; Hasegawa et al., 2013). Salinity affects at least 800 million ha, almost one-fourth of the world's arable land (Zhu 2016). Salt stress is mainly caused by sodium ions, a high concentration of which causes ion toxicity, osmotic stress, and an imbalance in the K^+^/Na^+^ ratio and has deleterious effects on critical biochemical processes (Gul et al., 2019). To cope with long-term salt stress, plants, in particular perennials, have evolved a range of intrinsic mechanisms and adaptive strategies. Transcriptional regulation networks control plant signaling pathways to regulate biological processes in response to salt stress (Saint André et al., 2021). These responses involve transcription factors (TFs), such as those of the *GRF*, *MYB*, NAM ATAF and CUC (*NAC*), and *WRKY* families (Ding et al., 2015a, 2015b; Fang et al., 2017; Mao et al., 2020). Expression of some TFs is induced by salt stress, which targets downstream stress-responsive genes such as ion transport and ATPase H^+^ (Mser et al., 2002; Yao et al., 2020).

The *GRF* family is a plant-specific TF family whose first member has identified 22 years ago (Zhang et al., 2008; Kim et al., 2010). GRF proteins harbor conserved QLQ and WRC domains in their N-terminal regions. The QLQ (glutamine, leucine, glutamine, and IPR014978) domain is accompanied by mostly bulky aromatic/hydrophobic amino acids (Zhang et al., 2008). These conserved regions may be important for the protein–protein interaction function of the QLQ domain (Van der et al., 2000). The WRC domain contains a functional nuclear localization signal and a DNA-binding motif (Kim and Tsukaya, 2015). The C-terminal regions of GRF proteins show marked sequence divergence but have common features reminiscent of TFs (Hewezi et al., 2012; Kim and Tsukaya, 2015). GRFs are reportedly involved in leaf and root development (Amin et al., 2015; Kim and Tsukaya, 2015). They may coordinate plant growth with defense signaling and stress responses and activate stress-related genes, increasing plant tolerance to salt stress (Liu et al., 2014; Kim and Tsukaya, 2015). For example, Arabidopsis (*Arabidopsis thaliana*) *atgrf7* mutants are more tolerant to salinity and drought stress than wild-type and *AtGRF7* overexpression lines (Kim et al., 2012). In addition, GRFs are important in biotic stress processes, as in the plant-parasitic cyst nematode *Heterodera schachtii*, and the parasite employs the plant's regulatory miR396/GRF module to control the initiation and subsequent maintenance phase of the syncytium in *Arabidopsis* (Liu et al., 2012). Thus, considering the growth-related functions of GRFs and their roles in abiotic and biotic stress responses (Kim and Tsukaya, 2015), GRFs, a small TF family, may appear to play a central role in coordinating plant growth and defense signaling. However, the upstream regulatory factors of GRF remain poorly understood, particularly in perennial plants.

Heterodimerization between TFs is widely associated with biological diversity and functional specificity for different metabolic processes in plants (Liu et al., 2022). Yeast two-hybrid-sequencing (Y2H-seq) analysis enables high-throughput investigation of protein–protein interactions and is a powerful tool in vitro due to its ability for large-scale screening of protein pairs (Chen et al., 2021; Niu et al., 2022). For example, yeast two-hybrid (Y2H) screening of ∼8,000 open reading frames (ORFs) generated an interactome map comprising 6,200 protein–protein interactions in *Arabidopsis* (*Arabidopsis* Interactome Mapping Consortium, 2011), and screening of ∼13,000 ORFs yielded a human interactome network consisting of 14,000 protein–protein interactions (Rolland et al., 2014).

*Populus*, a genus of woody perennial, includes rapidly growing woody plants long used as a model for studying angiosperm tree physiology and genetics. A common perennial deciduous plant in salinity zones, *Populus*, is resistant to high-salinity soils (Ma et al., 2013). Gene regulatory networks (GRNs) describe how genes interact with one another to regulate a function (Marshall et al., 2019). In *Populus*, environmental responses and complex developmental processes are controlled by various hierarchical GRNs consisting of TFs and stress-responsive genes (Kawaura et al., 2008; Ma et al., 2013). In this study, we constructed a GRN of the response of poplar 84 K (*Populus alba* × *Populus glandulosa*) to salt stress by combining time-course transcriptome analysis and unsupervised hierarchical clustering analysis. We reconstructed a growth-regulating factor 15 (PagGRF15)-mediated GRN by integrating target genes and interacting proteins identified by DNA affinity purification sequencing (DAP-seq) and Y2H-seq, respectively. Our findings provide insight into the popular regulatory network related to salt stress response and the role of *GRF* in the response to this stress.

## Results

### Physiological and transcriptomic response to salt stress

**Figure 1:**
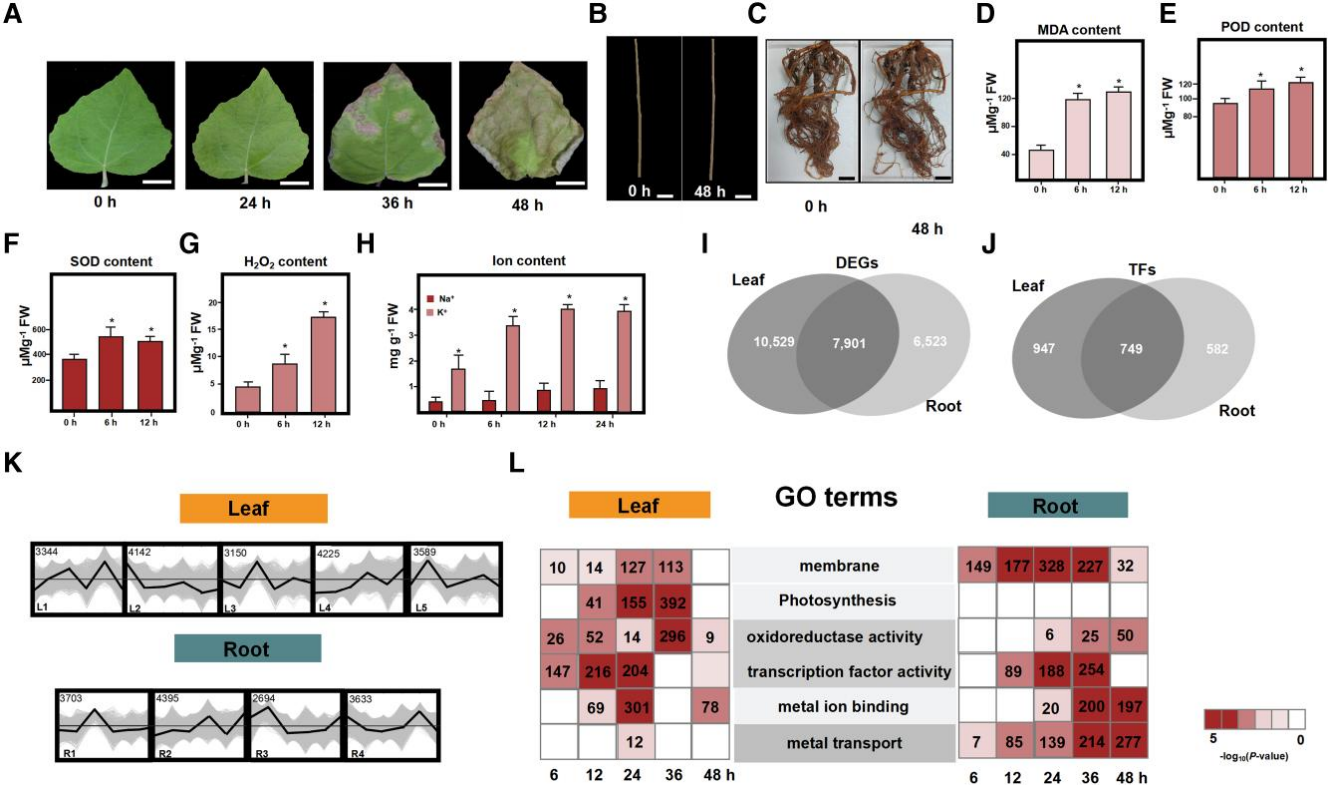
Temporal dynamics of poplar 84 K physiological indices and transcriptome during salt treatment. A, Phenotypic differences of leaf after 0, 24, 36, and 48 h of salt treatment. Bars, 2 cm. Phenotypic differences of stem (B) and root (C) after 0 and 48 h of salt treatment. Bars, 2 cm. MDA content (D), peroxidase (POD) activity (E), superoxide dismutase (SOD) activity (F), and H_2_O_2_ content (G) in leaves of poplar 84 K under salt stress. H, Na^+^ and K^+^ content of cuttings under salt stress. I, Venn diagrams of DEGs overlapping between leaf and root. DEG means differentially expressed gene. J, Venn diagrams of salt stress-responsive TFs overlapping between leaf and root. TF means transcription factor. K, Expression of nine salt-responsive gene clusters over time based on hierarchical clustering analyses between root and leaf. L, GO terms enriched in DEGs in root and leaf; red indicates significant enrichment (–log10 [*P*-value]). (*, *P* < 0.01, Student’s *t*-test).

Three-month-old cuttings of poplar 84 K were subjected to salinity shock (200 mM NaCl) for 48 h. After 24 h, most leaves wilted and detached (Figure 1A). The phenotype of stressed stems and their roots did not change substantially within 48 h (Figure 1, B and C). To assess the effects of salt stress on physiological activity, we examined the activity of the antioxidant enzymes superoxide dismutase (SOD) and peroxidase (POD), the malondialdehyde (MDA) content, H_2_O_2_ level, and the ion content in poplar under salt stress. In response to salt stress, SOD, POD, MDA, and H_2_O_2_ activities increased significantly from 6 to 12 h after treatment (189%, 249%, 164%, and 263%, respectively; Figure 1, D–G). The K^+^ content began to increase after 6 h of stress, and Na^+^ content began to increase after 12 h (Figure 1H). The reduced enzyme activity and ion toxicity resulted in severe physiological injury to the leaf (Figure 1, A–H).

To identify salt-responsive genes, we generated RNA-seq data from the leaf and root at six-time points. A total of 18,450 differentially expressed genes (DEGs) were obtained from the leaf, and 14,424 DEGs were obtained from the root (Table 1; Figure 1, I and J; Supplemental Tables 2–6). For leaf, gene ontology (GO) analyses of salt-responsive genes revealed 31 significantly (*P* < 0.001) enriched terms, including “photosynthesis,” “oxidoreductase activity,” “biosynthetic process,” “response to biotic stimulus,” and “transcription factor activity” (Supplemental Table 4). In the root, DEGs were related to “regulation of transcription,” “plant hormone signal transduction,” “metal transport,” “ribosome,” “phytohormone signaling,” “membrane,” and “RNA binding” (Supplemental Table 5). This indicates that leaf and root have different processes of adapting to salt stress.

**Table 1:** Genes differentially expressed in poplar 84 k in response to salt stress

Time of response	6 h	12 h	24 h	36 h	48 h
Total DEGs	7,671	13,360	5,505	8,718	11,158
Leaf	6,786	12,895	4,185	7,592	3,990
Root	979	2,831	3,287	2,674	10,171

To gain insight into the regulatory mechanism behind the salt stress response, we performed hierarchical clustering analyses of all DEGs. Five modules (L1, L2, L3, L4, and L5) were identified in the leaf, and four modules (R1, R2, R3, and R4) were identified in the root (Figure 1K). GO enrichment analyses of the coexpression clusters revealed that although multiple metabolic pathways were present in a single cluster, some clear patterns could be identified (Figure 1L). For example, in leaf, genes involved in “oxidoreductase activity,” “abiotic stress stimulus,” and “plant-pathogen interactions” were enriched in cluster L4 and exhibited an increasing trend from 0 to 12 h, indicating active reactive oxygen species (ROS) scavenging. Genes involved in “photosynthetic process” and “metabolic process” were enriched in cluster L2 and showed decreasing trends to 48 h (Figure 1L). These two GO terms are consistent with the physiological changes observed under salt stress, as shown that SOD, POD, and MDA activities increased significantly over the period under stress (Figure 1, D–G). In the root, genes involved in “protein processing in ER,” “metal transport,” and “transmembrane” were mainly enriched in cluster R3, which exhibited an increasing trend from 12 to 36 h, which indicates that membrane biosynthesis and ion transport pathways are enhanced in response to salt stress in root (Figure 1L).

### Identification of candidate TFs that respond to salt stress in poplar

TFs control plant signaling pathways to regulate many biological processes in response to salt stress (Wu et al., 2021b). In this study, 1,696 and 1,331 TFs were specifically expressed in leaf and root, respectively, at different stress response stages, and these TFs exhibited divergent expression patterns (Figure 1, J and K). Several TF families—including *ERF* and *GRF—*are involved in regulating salt stress response (Supplemental Table 7). Plant-specific TF GRF proteins are involved in responses to abiotic and biotic stresses, differentiation, development, metabolism, and defense (Kim and Tsukaya, 2015). In the coexpression network, seven GRF TF-encoding genes (*PagGRF*4, *PagGRF*7, *PagGRF*10, *PagGRF*11, *PagGRF*12, *PagGRF*15, and *PagGRF*16) were enriched in several temporally dynamic clusters (L1, L3, and R4). These clusters were enriched in “metabolic process,” “oxidoreductase activity,” “transcriptional regulatory”, and “metal transport.” Promoter enrichment revealed that DNA-binding motifs of GRF (TGTCA motif) were significantly enriched in the promoters of the L1, L3, and R4 clusters (*P* < 0.05; Supplemental Table 8), which suggests a role of GRFs as potential regulators of salt stress tolerance.

**Figure 2:**
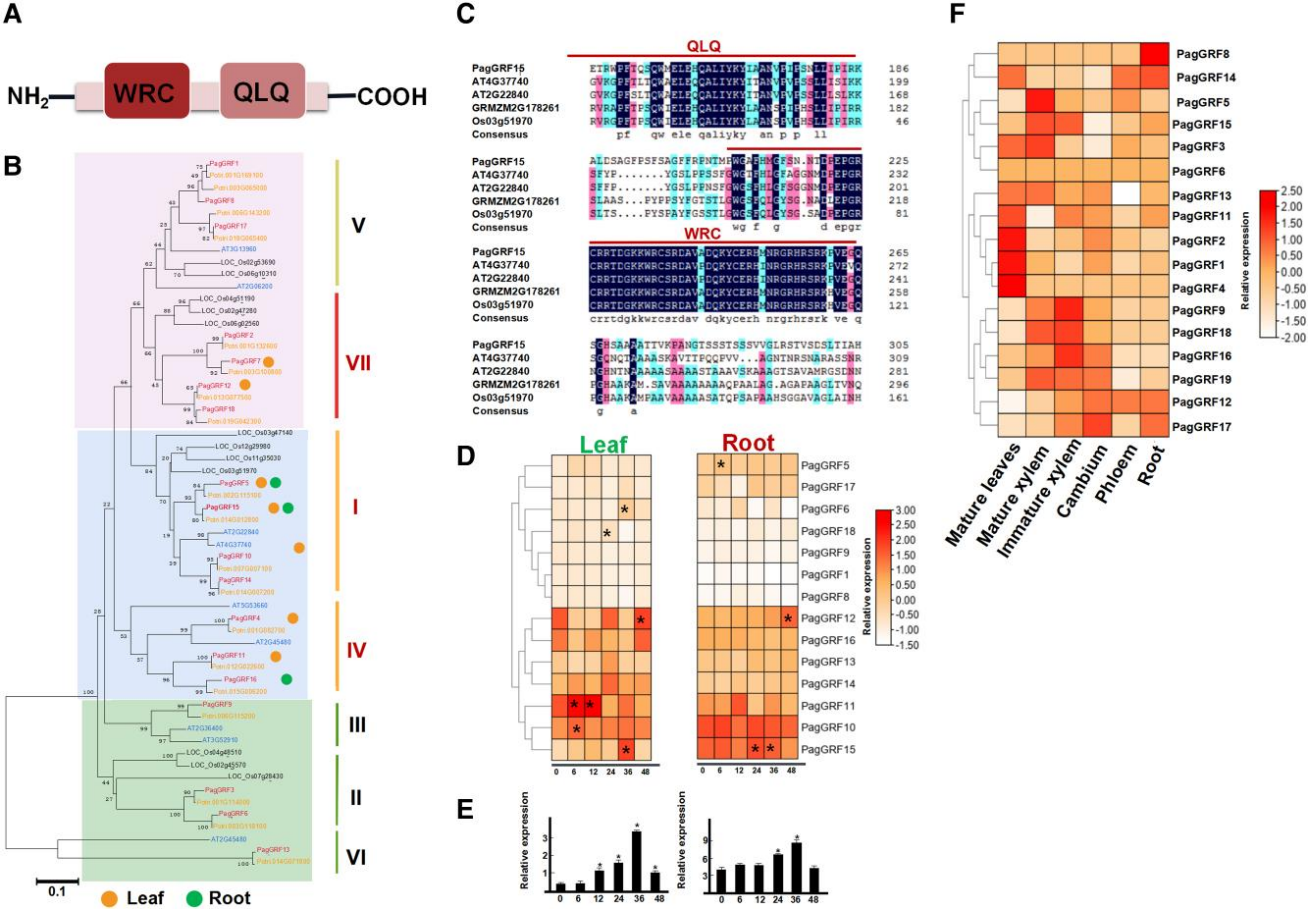
Multiple amino acid sequence alignment and phylogenetic tree of GRF proteins. A, Domain architecture of GRF. B, Phylogenetic analyses of GRF homologs from *P*. *trichocarpa*, *P*. *alba* × *P*. *glandulosa* 84 K, *Z*. *mays*, *O*. *sativa*, and *A*. *thaliana*. Genes indicated by yellow and green circles are salt responsive in the leaf and root, respectively. Bars = 0.1. C, Multiple alignment of the amino acid sequences of GRF proteins from poplar 84 K (*P*. *alba* × *P*. *glandulosa*), *Z*. *mays*, *O*. *sativa*, and *A*. *thaliana*. D, *PagGRF* expression in poplar 84 K leaf and root after salt stress for 0, 6, 12, 24, 36, and 48 h. Significant differences compared with the 0 h were determined using Student’s *t-*test: *P < 0.05. Values are means ± Se (*n* = 3). E, *PagGRF15* expression in poplar 84 K leaf and root after salt stress for 0, 6, 12, 24, 36, and 48 h, determined by RT-qPCR. Significant differences compared with the 0 h were determined using Student’s *t-*test: *P < 0.05. Values are means ± Se (*n* = 8). F, Expression of patterns of *GRF* genes within various tissues by RNA-seq including mature leaves, immature xylem, mature xylem, cambium, phloem, and root.

Multiple sequence alignment revealed that the amino acid sequences of the GRF proteins contained highly conserved domains (Figure 2A; Supplemental Table 2). GRF proteins were classified into subfamilies I and II to VII on the basis of the architecture and sequence of the domain (Figure 2B). Note that seven salt-responsive GRF TFs were classified into clades I (*PagGRF*10 and *PagGRF*15), IV (*PagGRF*4, *PagGRF*11, and *PagGRF*16), and VII (*PagGRF*7, and *PagGRF*12), which suggests roles for GRF subfamilies in the salt stress response (Figure 2C). It is worthy of note that the expression level of *PagGRF15* was significantly altered with an upward trend under salt stress in both leaves and roots (Figure 2D). To further explore the GRN of the salt stress response, we constructed a multilayer hierarchical GRN by combining all the differentially expressed TFs and PTGs (potential target genes) involved in the ion transporter, oxidoreductase activity, and photosynthetic pathway analysis described earlier (Supplemental Figure 1). As a result, a total of 21 TF-PTG pairs were identified as being located in the bottom, middle, and top layers of this network. We thus identified five key TFs upstream of salt stress-regulated genes, namely *GRF15*, *MYB65*, *ERF1,* and *bZIP4* (*P* < 0.05; Supplemental Figure 1). Collectively, these results indicate that the *PagGRF15* is a key and specific player in the basal salt stress response.

### Allelic variation of TFs/target genes in the GRN substantially affects growth and enzyme activity

To further explore the effects of salt-responsive TFs and DE-PTGs on phenotypic traits, single-nucleotide polymorphism (SNP)-based associations were performed using a mixed linear model in TASSEL v.5.2. Using the genomic resequencing data from 435 individuals, a total of 194,030 high-quality common SNPs (minor allele frequency >5%; missing data ≤10%) within 2,278 TFs genes (Supplemental Table 7) and 872 DE-PTGs which involved in the ion transporter, oxidoreductase activity, and photosynthetic pathway were identified (Supplemental Table 3), with the average frequency of occurrence of 19.41 kb^−1^.

Using the genomic resequencing data from 435 individuals, association analysis identified 46 significant SNP-trait associations with *P*-values ≤ 1.06 × 10^−5^ (threshold = 1/*n*), representing 39 unique SNPs associated with 17 phenotypic traits, located in *GRF15* and seven DE-PTGs (Supplemental Table 16). Each SNP explained 1.98%–21.22% of the phenotypic variance (*R*^2^); the most significant association was between GRF15_SNP32 and cyto-FBP (*P*-value = 2.32 × 10^−6^), which accounted for 13.51% of the variation (Supplemental Table 16). We also found that *GRF15* and *HAK6* were associated with Rubisco (Ribulose bisphosphate; Supplemental Figure 2A), cytoplasm (cyto-FBP; Supplemental Figure 2A), and LFNR (NADP ^+^ oxidoreductase; Supplemental Figure 2A). Significant SNPs associated with multiple traits showed disparate genetic effects; for instance, the SNPs GRF15_SNP15, GRF_SNP58, and HAK6_SNP53 were associated with rubisco activase (RCA), and the SNPs GRF15_SNP11 and HAK6_SNP43 were associated with Rubisco, with either additive or dominant effects (Supplemental Table 16). Notably, GRF15_SNP11, HAK6_SNP19, HAK6_SNP43, and HAK6_SNP53 are located in the 3′ untranslated regions (UTR) of *GRF15* and *HAK6*, and GRF15_SNP32 and HAK6_SNP11 are located in the intron and upstream of *GRF15* and *HAK6,* respectively (Supplemental Table 16). We constructed a network based on the epistatic SNP–SNP interactions within PTGs and genes (Supplemental Figure 2). In this network, HAK6 and GRF15 formed a pairwise epistatic interaction that affected RCA, Rubisco, and cyto-FBP (Supplemental Figure 2B).

### PagGRF15 promotes leaf development and Na *^+^* efflux

**Figure 3:**
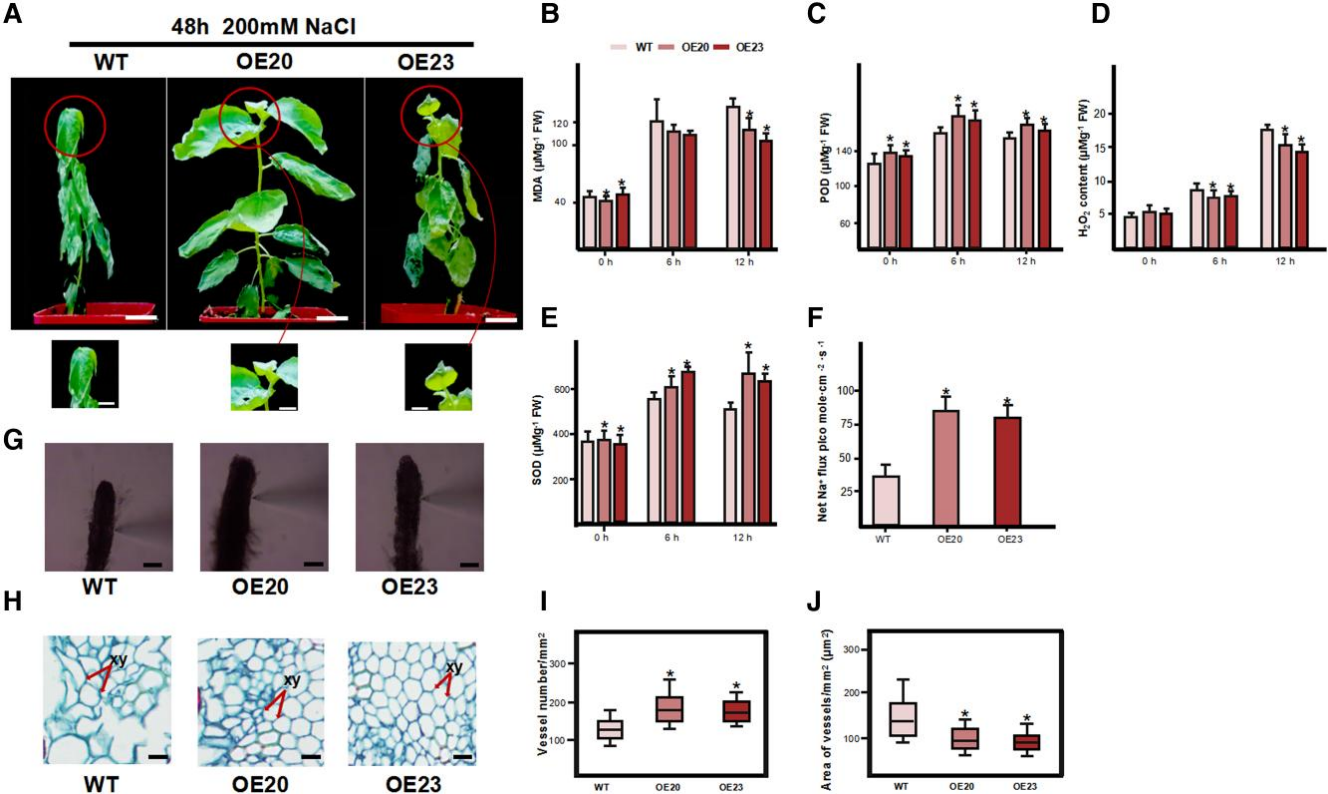
35S:*PagGRF15* poplar lines show tolerance to salt stress. A, Phenotypic differences in short-term salt treatment. Bars, 2 cm. The individual images were digitally extracted for comparison. MDA content (B), peroxidase (POD) activity (C), H_2_O_2_ content (D), and SOD activity (E) in leaves of WT and 35S:PagGRF15 (OE) plants under normal and salt stress conditions. *Significant difference from the WT at *P* < 0.01 by Student’s *t*-test. F, Net Na^+^ flux in the root meristem zone of WT, OE20, and OE23 by NMT. Two-month-old cuttings were pretreated with 100 mM NaCl for 24 h. *Significant difference from the WT at *P* < 0.01 by Student’s *t*-test. G, Phenotypic difference in root apical tissue in 100 mM NaCl for 24 h. Bars, 0.1 cm. H, Root cross-sections of WT and OE-PagGRF15 transgenic plants. Bars, 25 µm. xy, xylem. I, Number of vessels per cross-sectional area (mm^2^). J, Area of vessels (µm^2^) per cross-sectional area (mm^2^). *Significant difference from the WT at *P* < 0.01 by Student’s *t*-test. Values are means ± Se (*n* = 20). In boxplot, center line: the value in the middle after the data is sorted from small to large; box limits: Maximum and minimum of the value; upper (Q2) and lower (Q1) quartile mean number at 75% of the data series and number at 25% of the data series; 1.5× interquartile range: = Q3–Q1. Bottom edge and upper edge mean Q1–1.5*IQR and Q3 + 1.5*IQR, respectively.

To investigate the function of *PagGRF*15 in the salt stress response, we generated transgenic lines of poplar 84K overexpressing *PagGRF15* named OE20-*PagGRF15* and OE23-*PagGRF15*. Compared with the WT, the OE20 and OE23 lines had a significantly larger leaf area increased by 53.3% and 35.1%, respectively (Supplemental Figure 3; Table 2), which is consistent with a previous study (Wu et al., 2021a, 2021b). Furthermore, the fresh weight (FW) of OE20 and OE23 plants was 57.8% and 64.2% higher, respectively, than the WT, and the dry weight (FW) of OE20 and OE23 plants was 27.7% and 23.1% heavier, respectively, than the WT under normal condition (Supplemental Figure 3). After salt stress, most leaves of WT plants wilted and detached, whereas those of OE plants remained turgid. POD and SOD activities of OE plants were significantly enhanced by 37.3% and 55.0%, respectively (Figure 3, C and E), whereas the MDA and H_2_O_2_ contents decreased by 18.6% and 36.9% compared with the WT (Figure 3, B and D). Therefore, overexpression of *PagGRF15* enhances tolerance to salt stress by activating the antioxidant system (Figure 3).

**Table 2:** Vessel number/mm^2^, area of vessels/mm^2^ (µm^2^), and leaf area (cm^2^) of 3-month-old PagGRF15 overexpression transgenic lines

Sample	Vessel number/mm^2^	*P*-value	Area of vessels/mm^2^ (µm^2^)	*P*-value	Leaf area (cm^2^)	*P*-value
WT	82.87 ± 34.01		141.09 ± 54.64		44.03 ± 6.15	
OE20-PagGRF15	149.31 ± 50.64	0.00527	76.84 ± 30.51	0.00916	67.87 ± 7.89	0.0195
OE23-PagGRF15	142.6 ± 67.09	0.00218	80.1 ± 25.25	0.00462	59.12 ± 6.61	0.0315

Values are means ± Sd. *P*-value was calculated by *t*-test between OE and WT lines. *n* = 20.

The plasma membrane (PM) Na^+^ efflux ratio and root-to-shoot Na^+^ translocation are important for alleviating salt stress. We measured Na^+^ efflux in the meristem zone of 3-month-old WT and OE plants following treatment for 24 h with 100 mM NaCl. Salt-stressed root exhibited a pronounced net Na^+^ efflux that was significantly (2.41-fold) higher in the OE than WT plants (Figure 3F), which indicates that *PagGRF15* overexpression promotes proton pumping activity under salinity stress. In addition, the root vessels of OE plants were smaller than those of WT plants (Figure 3, H and J), and the vessel number per unit area of OE plants was 2.32-fold of WT plants (Figure 3I). Tissue specificity analysis revealed that *PagGRF15* was expressed in the root and xylem tips under control culture conditions (Figure 2, E and F). As a result, the increased number of shorter fiber cells and enhanced vessel development could be attributable to decreased root-to-shoot Na^+^ translocation via xylem. Therefore, overexpression of *PagGRF15* not only increases poplar growth but also enhances its tolerance to salt stress (Figure 3A).

### PagGRF15 interacts with a set of phytohormone-related proteins

**Figure 4:**
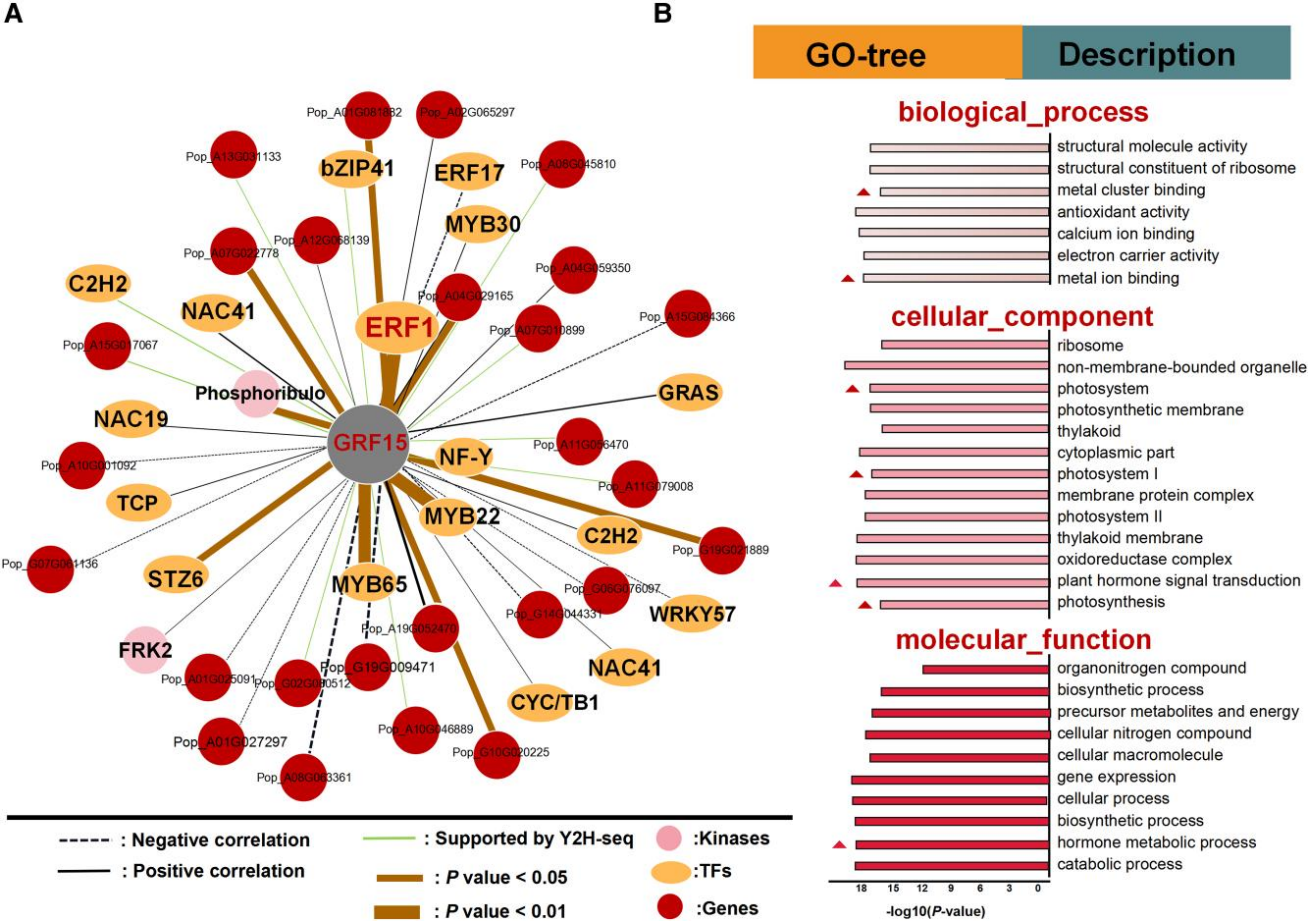
PagGRF15 interacts physically with a number of proteins. A, PagGRF15 collaborative network based on Y2H-seq and the coexpression connectivity matrix of salt stress-related proteins, including STZ6 and ERF1. The black dotted and solid lines represent the negative and positive correlation respectively, supported by correlated coexpression. The green solid lines represent the correlation only supported by Y2H-seq. The brown solid lines represent the correlation supported by both Y2H-seq and correlated coexpression, and the thickness of the line represents the size of the *P*-value. TF mean transcription factor. B, GO enrichment analyses of PagGRF15-interacting proteins. GO means gene ontology annotation.

Heterodimerization, which is associated with biological diversity, is functionally specific to different metabolic processes in plants (Liu et al., 2022). To identify PagGRF15-interacting proteins, we performed Y2H screening using a cDNA library with PagGRF15 as the bait. A total of 420 non-redundant potential interacting proteins of PagGRF15 were identified, including nine TFs (*C2H2*, *MYB*, *TCP*, *AP2/ERF*, and *NF-Y*), five kinases (phosphoribulo, lectin, and shikimate kinases), as well as several enzymes and RNA-binding proteins (Supplemental Figure 4 and Table 10). Moreover, 19.53% of the interacting protein genes correlated positively with *PagGRF15* (*R*^2^ > 0.30, *P* < 0.05; Supplemental Table 13) under salt stress and were related to “hormone-mediated signaling pathway,” “hormone metabolic process,” “photosynthesis,” and “cation binding” (Figure 4B). Therefore, *PagGRF15* may recruit phytohormone-related proteins under salt stress. In total, 31 genes were mapped into the “hormone-mediated signaling pathway,” among which *ERF1* bound to a GCC box ethylene-responsive element and was induced by salt stress, similar to *PagGRF15* (*R*^2^ = 0.43, *P* < 0.05; Supplemental Table 13). Therefore, there is a strong interaction between PagERF1 and PagGRF15 (Figure 4A).

The interaction in vitro was further verified by point-to-point assays through Y2H (following yeast liquid dilution concentration as control: 10^0^, 10^−1^, and 10^−2^) and bimolecular fluorescence complementation (BiFC) assays in vivo (Supplemental Figure 5). As a result, it appears conceivable that PagERF1 and PagGRF15 form heterodimers and play an important role in salt stress response by regulating the expression of some other salt-responsive genes. Such a notion has prompted us to investigate the regulatory specificity of the PagERF1-PagGRF15 heterodimer by identifying the immediate target genes of PagGRF15.

### *PagGRF15* directly regulates expression of the salt-responsive gene high-affinity K^+^ transporter 6 (*PagHAK*6)

**Figure 5:**
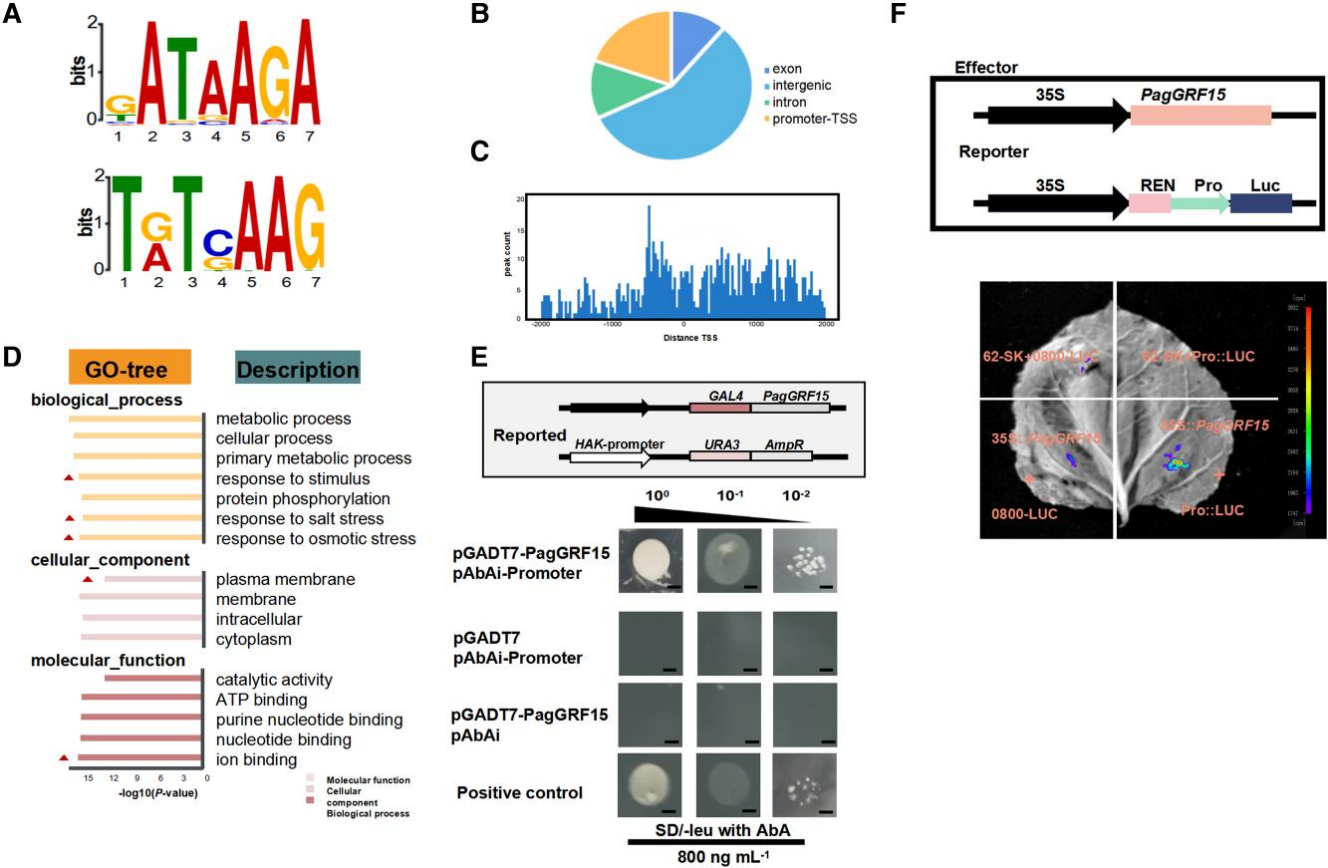
Genome-wide identification of PagGRF15 binding sites and motifs. A, De novo motif discovery with MEME-ChIP revealed that GATAAGA and TGTCAAG are enriched at PagGRF15 binding sites. B, Relative distribution of binding peaks across genomic regions. C, Distribution of the first occurrence of GATAAGA and TGTCAAG motifs as a function of distance from the peak summits of binding sites. TSS means transcription start site. D, GO enrichment analyses of 570 target genes of PagGRF15. GO means gene ontology annotation. E, Y1H analyses of the interaction between PagGRF15 and PagHAK6 promoters. Top, reporter and effector vectors. Reporter and effector constructs were co-transformed into yeast Y187 cells, and positive transformants were identified by spotting serial dilutions onto SD/-Leu medium supplemented with 800 ng mL^−1^ AbA. pGADT7-53 + pAbAi-Lam and pGADT7 + pAbAi-promoter were constructed in the same manner as the positive and negative controls, respectively. Yeast dilution factors: 10^0^, 10^−1^, and 10^−2^ (left to right). Bars, 0.25 cm. F, Dual-luciferase reporter analysis of *PagGRF15* activating the *PagHAK6* promoter in *N. benthamiana* leaves. Schematic of the reporter and effector constructs is shown at the top. The effector and reporter vectors were instantaneously co-transformed into leaves of *N. benthamiana* and cultured in a greenhouse under control conditions for 48 h (light culture for 24 h, dark culture for 24 h). The experiments were performed three times, and similar results were obtained.

We performed DAP-seq to identify genes directly targeted by PagGRF15 as described by Bartlett et al. (2017). Analyses of the sequencing data revealed 2,238 putative target sites bound by PagGRF15 in vitro (Supplemental Table 11). The majority (61.83%) of the peaks identified were located in proximal upstream regions, including in the 5′-untranslated region (5′-UTR) and around the transcription start site (Figure 5, B and C; Supplemental Figure 7 and Table 11). Of these 2,238 sites, 570 putative target genes (*Q* < 0.05) in the poplar genome had promoter sequences enriched with GATAAGA and TGTCAAG motifs (*P* < 0.05; Figure 5A). The putative target genes were related to “cytokinin catabolic process” (GO 0009823), “response to salt stress” (GO 0009651), “response to stimulus” (GO 0050896), “plasma membrane” (GO 0005886), “cellular anatomical entity” (GO 0110165), and “ion transmembrane transporter activity” (GO:0015075). A total of 27 genes were mapped to the transporter activity process, including K^+^ potassium transporter 6 (HAK6) and amino acid transmembrane transporters (AAPs; Figure 5D). HAK may mediate K^+^ absorption by the PM and promote the maintenance of Na^+^/K^+^ homeostasis under salt stress. Note that the expression of *PagHAK6* was induced by salt stress and showed a similar trend as *PagGRF15* (*R*^2^ = 0.31, *P* < 0.05; Supplemental Table 12). Furthermore, PagHAK6 and other marker genes’ expression was strongly enhanced in OE lines but was suppressed in WT lines under control and salt conditions (Supplemental Figure 5C), which indicates that PagGRF15 regulates genes involved in ion transporter pathways under salt stress. Note that the expression of *PagHAK6* was induced by salt stress and showed a similar trend as *PagGRF15* (*R*^2^ = 0.31, *P* < 0.05; Supplemental Table 12). To confirm the binding of *PagGRF15* to the promoter region of *PagHAK6*, we used regions of the promoter to drive an *AUR1-C* reporter gene in a yeast one-hybrid assay (Y1H) and luciferase reporter assays (LRA) to further verify this result. *PagGRF15* interacted stably in the proximal promoter region of *PagHAK6* (Figure 5, E and F). Several other growth and development biological processes were enriched among the target genes (Supplemental Table 12), such as cytokinin oxidase/dehydrogenase (CKX), which enhances the meristematic potential of formative cells during leaf development (Higuchi et al., 2004).

To explore the effects of target genes on phenotypic traits, we performed single SNP-based association analyses. Among the 164 significant SNP-trait associations, 67 common SNPs in 31 target genes correlated with associated traits with disparate genetic effects. For example, *HAK6*_SNP41 was associated with transpiration rate (additive effect, *R*^2^ = 11.26%), photosynthesis (dominant effect, *R*^2^ = 11.19%), and leaf length (additive and dominant effects, *R*^2^ = 12.24%), which indicates that *PagHAK6* has additive and/or dominant effects on growth and stress responses (Supplemental Table 16). These results suggest that *PagGRF15* regulates salt-responsive genes by modulating transporter activity and may systemically influence the expression of multiple growth-related genes.

### Overexpression of *PagHAK6* reduces H_2_O_2_ and increases plant growth under salt stress

**Figure 6:**
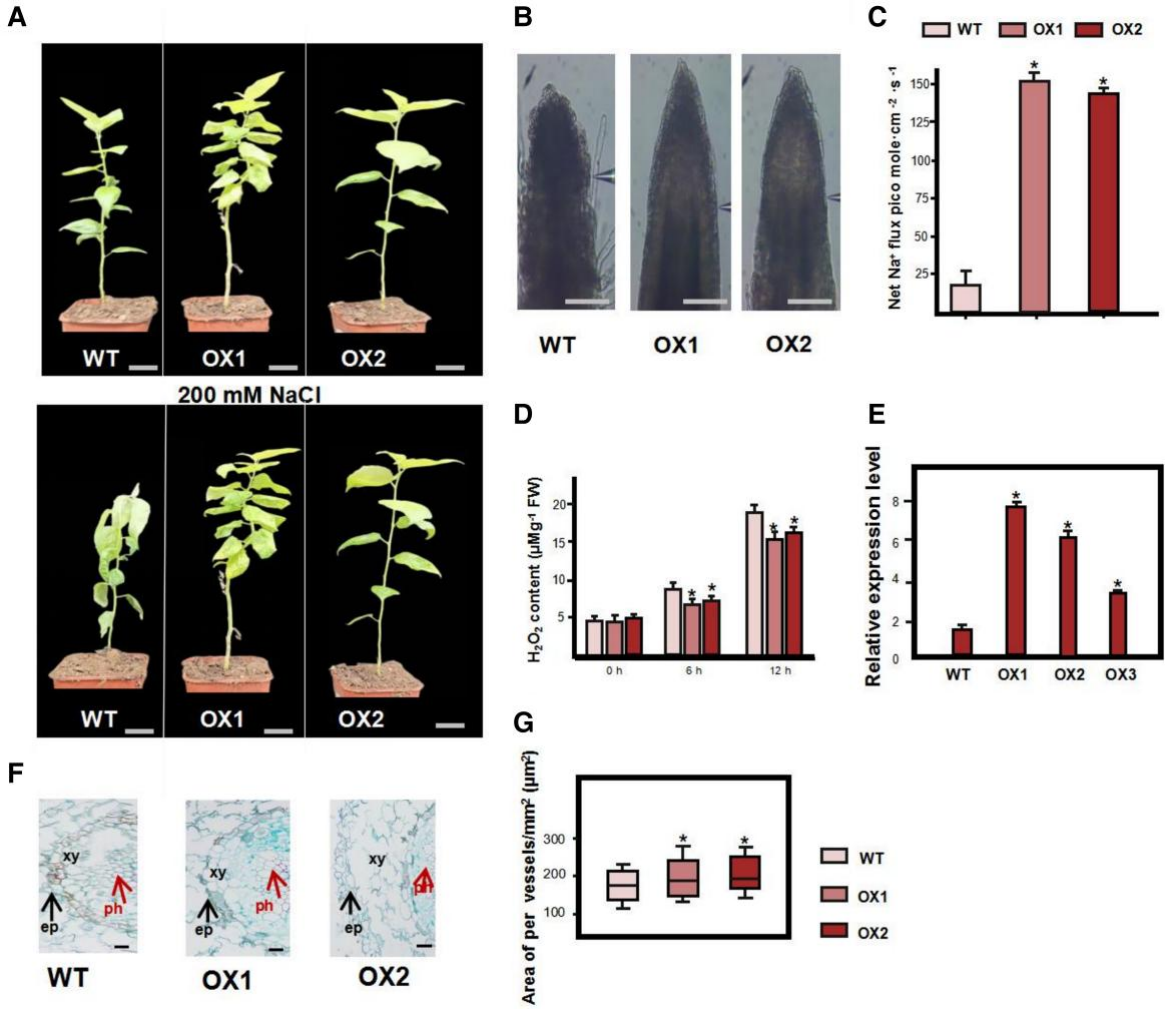
Enhanced salt tolerance in transgenic poplar overexpressing PagHAK6. A, Phenotypes of WT and transgenic (OX) plants under salt stress (200 mmol NaCl). B, Phenotypic difference in root apical tissue in 100 mM NaCl for 24 h. Bars, 0.1 cm. C, Net Na^+^ flux in the root meristem zone of WT, OX1, and OX2 by NMT. Two-month-old cuttings were pretreated with 100 mM NaCl for 24 h. *Significant difference from the WT at *P* < 0.01 by Student’s *t*-test. D, H_2_O_2_ content in leaves of WT and 35S:PagHAK6 plants under normal and salt stress conditions. E, RT-qPCR of three HAK6-transformed poplar lines (OX). *PagActin* was used as a reference for normalization. Values are means ± Se (*n* = 20). *Significant difference from the WT at *P* < 0.01 by Student’s *t*-test. F, Root cross-sections of WT and OE-PagHAK6 transgenic plants. Bars, 25 µm. ph, phloem; xy, xylem; ep, epidermis. G, Number of vessels per cross-sectional area (mm^2^). *Significant difference from the WT at *P* < 0.01 by Student’s *t*-test. In boxplot, center line: the value in the middle after the data are sorted from small to large; box limits: Maximum and minimum of the value; upper (Q2) and lower (Q1) quartile mean number at 75% of the data series and number at 25% of the data series; 1.5× interquartile range: = Q3–Q1. Bottom edge and upper edge mean Q1–1.5*IQR and Q3 + 1.5*IQR, respectively.

HAK, as a recently identified Na ^+^ -selective transporter, confers vital roles in promoting shoot Na^+^ exclusion (Zhang et al., 2019). The phylogeny tree indicated that these members were grouped into four clusters (clusters I–V), and *PagHAK6* fell into cluster IV (Supplemental Figure 9B and Table 14). We first analyzed the subcellular localizations of PagHAK6 protein, and PagHAK6 was observed in the membrane of *N. benthamiana* (Supplemental Figure 10). To confirm the function of the target gene *PagHAK*6 in salt tolerance, we generated transgenic poplar overexpressing *PagHAK6*. The transgenic lines (OX1 and OX2) with high expression were selected for further study (Figure 6E). After salt stress, most leaves and roots of WT plants wilted and detached, whereas those of OE plants remained turgid (Figure 6). We measured Na^+^ efflux in the meristem zone of 3-month-old WT and OX plants following treatment for 24 h with 100 mM NaCl. Salt-stressed root exhibited a pronounced net Na^+^ efflux that was significantly (6.28-fold) higher in the OX than in the WT plants (Figure 6C), which indicates that *PagHAK6* overexpression also promotes proton pumping activity under salinity stress. To investigate the effects of overexpression of *PagHAK6* on oxidative damage under salt stress, we performed H_2_O_2_ histochemical (DAB) staining. The H_2_O_2_ levels of transgenic poplars were similar under controlled growth conditions. However, after exposure to salt stress (200 mM NaCl) for 1 h, staining was increased in the leaves of the WT and OX1/2, but the leaves of transgenic poplars showed fewer brown spots than the WT (Supplemental Figure 8, E and F). Furthermore, the H_2_O_2_ content decreased by 21.4% compared with the WT (Figure 6D). Therefore, overexpression of PagHAK6 enhances tolerance to salt stress by activating the antioxidant system (Figure 6).

To explore whether *PagHAK6* affects growth and development under salt stress, we measured rates of increase in stem length (SL), FW, and dry weight (DW). No significant differences were observed in plant biomass between the OX and WT plants under normal conditions (Supplemental Figure 8A). However, after 15 days of salt stress, the SL of OX1 and OX2 plants was 59.2% and 46.1% higher, respectively, than that of the WT (Supplemental Figure 8B), and the FW of OX1 and OX2 plants was 96.3% and 78.9% heavier, respectively, than that of the WT (Supplemental Figure 8, C and D). In summary, under long-term salt stress conditions, the OX lines exhibited markedly higher increases in plant height and biomass. Therefore, OX-*PagHAK6* plants enhance salt tolerance by activating the antioxidant system and maintaining ROS homeostasis, thereby increasing biomass under salt stress.

## Discussion

### Identification of TFs and TFBSs responsive to salt stress

In light of the comprehensive analysis of the physiological index and transcriptomes of different tissues and stages, it becomes apparent that salt stress substantially induces DEGs in poplar leaf and root tissues. Here, 24,953 DEGs were categorized into nine clusters, and most of the physiological indices are well in line with the gene expression patterns (Figure 1, A–H). These large-scale changes likely include diverse salt-responsive TFs that activate or repress other salt-responsive genes (Reményi et al., 2004; Song et al., 2016; Vihervaara et al., 2018). We identified a number of TFs whose encoding genes exhibited significantly increased transcript abundance under salt stress, including a subset activated early or late after exposure to stress in leaf and root, probably because of different adaptive mechanisms (Supplemental Table 7).

There were links between upregulated TF genes and some TFBSs enriched in their promoters. Among the salt-upregulated genes, GRF TF genes and several GRF-binding sites (TGTCA motif) showed significant enrichment (Kim and Tsukaya, 2015; Wu et al., 2021a), consistent with motif enrichment by DAP-seq and enrichment of salt-responsive ERF TF genes and ERF-binding sites (GCC motif; Yu et al., 2016; Yao et al., 2020). Therefore, the evidence makes GRFs strong candidates for the regulator of salt stress response. Note that these TFs are also associated with the salt stress response in *A*. *thaliana* (Wu et al., 2021b).

*GRF* genes mediate leaf formation and growth in plants (Kim and Tsukaya, 2015; Wu et al., 2021a) and are implicated in responses to heat and drought stress (Khadiza et al., 2017; Zhao et al., 2021). In our study, *GRF* genes are capable of causing a stress-tolerance response under salt stress. In *A. thaliana*, GRFs are also involved in regulating comparable temporal patterns of downstream stress-responsive genes (Kim and Tsukaya, 2015), suggesting that GRF orthologues probably confer a conserved salt-tolerant mechanism in the two main dicotyledons and probably other plant species (Figure 2). Therefore, GRF TFs are implicated in developmental and physiological adaptations to salt stress.

### PagGRF15 implicated in salt stress tolerance and plant growth

**Figure 7:**
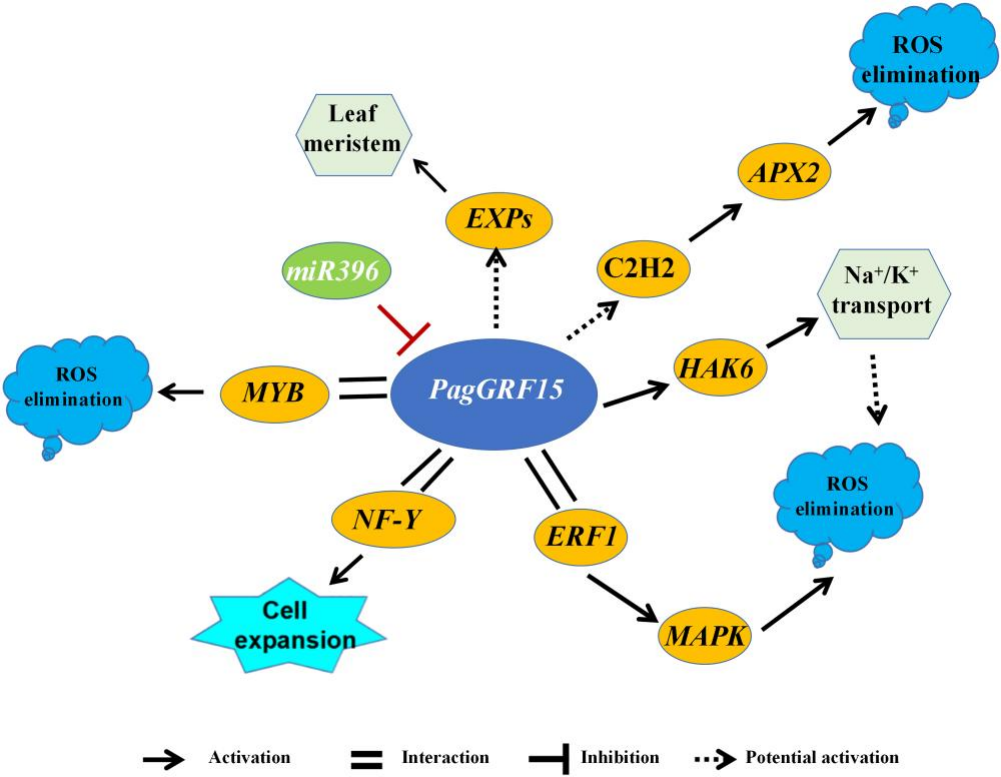
Proposed role of *PagGRF15* in tolerance to salt stress. Each solid line denotes a direct regulatory relationship reported previously or determined in this study (Y1H, degradome-seq, DAP-seq, and Y2H-seq); each dashed line denotes a potential regulatory relationship predicted by combining TRN and DAP-seq. ROS means reactive oxygen species.

The maintenance of plant morphology, biomass, and yield during salt stress depends on several physiological factors and internal mechanisms, such as ion transport, ROS scavenging, and the synthesis of protective proteins (Allakhverdiev et al., 2008; Ashraf and Harris, 2013; Teskey et al., 2015). In this study, Na^+^ efflux increased 63% in OE-*PagGRF15* compared with WT plants. Salt-tolerant *Populus* has permanently activated *HAK*, which is responsible for Na^+^ exclusion and compartmentalization (Ma et al., 2013) and promotes vacuolar Na^+^ sequestration (Filipa et al., 2009). The salt overly sensitive pathway including PM Na^+^/H^+^ antiporter, which is conserved in plants, regulates sodium ion homeostasis under salt stress. According to DAP-seq, LRA, and Y1H assays, *PagGRF15* bound to the TGTCAAG motif in the promoter and activated expression of *PagHAK6*. PagHAK6 orthologues with the same domain were identified in most of the other plant species, and they show 51%–89% similarities in sequence to PagHAK6 (Supplemental Figure 9, A and C), suggesting that PagHAK6 putative orthologues probably confer a conserved salt-tolerant mechanism. In rice (*Oryza sativa*), a *HAK6* homolog, *OsHAK5*, is involved in K^+^ uptake by root and K^+^ transport from roots to shoots under K ^+^ -deficient conditions (Yang et al., 2014). In poplar 84 K, tissue specificity analysis revealed that *PagHAK6* mainly was expressed in the root and xylem tips under control culture conditions (Figure 2F) and *PagHAK6* also correlated positively with the expression level of *PagGRF15* and *PagERF1* in different tissues (Supplemental Table 9). Transgenic lines overexpressing *PagHAK6* show increased plant biomass and ROS elimination under salt stress, and exhibited similar phenotypes with the *PagGRF15* OE plants. Therefore, overexpression of *PagHAK6* promotes maintenance of ROS homeostasis by enhancing control of K^+^/Na^+^ under salt stress, but not enhanced antioxidant compounds and antioxidant enzyme activity (Figure 6; Supplemental Figure 6A). Thus, we propose that the *GRF15*-*HAK6* module acts as a major positive transcriptional regulation module in the response to salt stress (Figure 7).

Excess ROS, such as those produced by salt stress, lead to oxidative conditions that are harmful to cells (Kuge et al., 2010; Wang et al., 2008). Overexpression of *PagGRF15* reduced H_2_O_2_ and MDA levels compared with control plants under salt stress, which suggests that Pag*GRF15* regulates genes involved in ROS homeostasis. In this study, many target genes in the oxidation-reduction process were enriched under salt stress. For example, *PagC2H2* was an upregulated target gene of *PagGRF15* (Supplemental Figure 6B). In *Populus trichocarpa*, the C2H2-type zinc finger protein ZFP1 enhances tolerance to salt and drought stresses by maintaining homeostasis of intracellular osmotic pressure and activating the antioxidant system (Liu et al., 2015; He et al., 2018). Our computational analysis with psRNATarget showed that PagGRF15 is targeted by miRNA396a in poplar with high affinity and we validated this interaction with a degradome sequencing (Figure 7; Supplemental Table 15). These results indicate that *PagGRF15* regulates ROS via *PagC2H2* under salt stress, which suggests a divergent regulatory chain; this needs to be verified by further research (Figure 7).

GRF regulates plant growth and development in annual herbs (Kim and Tsukaya, 2015). In poplar 84K, vessel diameter and leaf area increased 131% and 44%, respectively, in the *PagGRF15*-OE line compared with the WT, enhancing photosynthesis and Na^+^ translocation through the phloem to roots (Berthomieu et al., 2003; Ren et al., 2005; Sunarpi et al., 2005). *PagGRF15* directly regulated cytokinin oxidase/dehydrogenase (*PagCKX*; Supplemental Table 11), enhancing xylem development and cell division in leaves of OE plants, in agreement with prior reports that endogenous cytokinins in xylem sap are present in relation to leaf senescence (Wu et al., 2021a). Thus, unlike other stress tolerance genes such as *DREB2C* or *AtSAP5* (Lim et al., 2007; Hozain et al., 2012), overexpression of *PagGRF15* promotes plant growth and salt stress tolerance. Association analyses showed that *PagGRF15* and its target genes had mixed effects on phenotypic traits under salt stress, which also validates such a supposition (Supplemental Table 16).

### Dimerization of PagGRF15 with a series of TFs mediates transcriptional activation

The previous study indicated PpnGRF5-1 interacts with PpnGIF in poplar and ameliorates its regulatory diversity and capacity (Wu et al., 2021a). The recent advent of Y2H-seq with next-generation sequencing techniques provides a convenient and reliable method that can be used to supplement with classic Y2H approach including low plasmid extraction (Chen et al., 2021; Niu et al., 2022). Y2H screening identified a series of putative PagGRF15-TF interactions, including with C2H2, MYB65, AP2, and ERF1 TFs, providing insight into regulatory mechanisms associated with the salt stress response (Supplemental Table 10). *PagGRF15* and *PagHAK6* correlated positively with *PagERF1* in response to salt stress (*P <* 0.05). The ERF-binding motif (GCC box) in the 1 kb promoter region of *PagHAK6* indicates that *PagGRF15* may be recruited by *PagERF1*. *PagERF2* also interacted with *PagGRF15* (Supplemental Table 10), and we used regions of the promoter to drive an *AUR1-C* reporter gene in Y1H and LRA to further verify this result (Supplemental Figure 11). The two *PagERF* members belong to one subgroup as phylogenetically paired homologs (Toshitsugu et al., 2006; Zhuang et al., 2008) and participate in responses to high temperature, salt, cold, and drought stresses by reducing MDA and ROS accumulation (Schmidt et al., 2013; Wang et al., 2012). The *PagGRF15*–*PagERF1*–*PagHAK6* pathway is a component of the complex regulatory network of the plant salt stress response. *C2H2* and *MYB65* also interact directly with PagGRF15 and are key factors in the TF-mediated ROS scavenging regulatory pathway in plants (Laura et al., 2007; He et al., 2018), suggesting a deeper and wider biological network than expected.

All in all, this study on *Populus* salt-response GRNs revealed that trans-regulators commonly exist and the molecular regulatory mechanisms that underpin these functions remain largely elusive. The hypothesized working model that PagGRF15 and numerous interactive partners act in concert to confer salt stress tolerance may not only facilitate a chassis for further investigation but also offer a suite of candidate genes for the genetic improvement of salt tolerance in *Populus* and other crop plants.

## Materials and methods

### Plant materials and salt treatment

Three-month-old poplar clone 84 K (*P*. *alba* × *P*. *glandulosa*; Pag) cuttings were grown on a seed plot (15.0-h light [06:00–21:00], 22°C–25°C) in a greenhouse at Beijing Forestry University, Beijing, China (40°000 N, 116°200 E). The plants were watered with 1 L Hoagland nutrient solution every 2 weeks for 2 months before treatment. Relative humidity was maintained at 70%. For salt treatment, 36 uniform cuttings were subjected to moderate salt stress by watering with 2 L NaCl (200 mM). Total RNA was extracted from the roots and mature upper leaves (leaf numbers 4–20 from the shoot apex) 0, 6, 12, 24, 36, and 48 h after salt treatment. At each sampling, three biological replicates per treatment were used for phenotypic measurements, RNA-seq, and RT-qPCR.

### H_2_O_2_ and antioxidant enzyme assay

Poplar 84 K leaves were immediately frozen in liquid nitrogen for the determination of pigment content and enzyme activity. Briefly, 0.1 g samples of leaves were frozen in liquid nitrogen, homogenized in ice-cold 0.01 M phosphate buffer (1.5 mL, pH 7.2) and centrifuged at 14,000 g for 10 min at 4°C. The supernatant was used to measure SOD activity with a Plant SOD Assay Kit (Nanjing Jiancheng Bioengineering Institute, Jiangsu Province, China). First, 1.5-mL reaction buffer (0.013 M met, 6.3 × 10^−6^ M nitro blue tetrazolium chloride, 6.5 × 10^−6^ M riboflavin, 1 × 10^−4^ M EDTA, 0.05 M phosphate buffer, pH 7.8) was added to the supernatant followed by incubation at 25°C for 30 min, and absorbance at 560 nm was measured with a spectrophotometer. MDA activity was determined with a Catalase Assay Kit (Nanjing Jiancheng Bioengineering Institute) following the manufacturer's protocol. Leaf samples were ground in a microcentrifuge tube fitted with a plastic pestle containing ice-cold 0.01 M phosphate buffer (pH 7.2) with 1.13-mg dithiothreitol. The suspension was centrifuged at 14,000 g for 10 min at 4°C. We assayed the supernatant for MDA activity by measuring the linear rate of decrease in absorbance at 240 nm with a spectrophotometer. For H_2_O_2_ and POD activity, 0.1-g samples of leaves were homogenized in 1.5-mL ice-cold 0.01 M phosphate buffer (pH 7.2) for 30 min and centrifuged at 14,000 g for 10 min at 4°C. A Plant H_2_O_2_ and POD Assay Kit (Nanjing Jiancheng Bioengineering Institute) was used to measure H_2_O_2_ and POD activities in the supernatant. The supernatant was added to a mixture of 0.5 mL 0.8% (w/v) H_2_O_2_, 0.5 mL 0.1 M phosphate buffer, and 0.5 mL 0.1 M guaiacol buffer and incubated at 30°C for 8 min. Absorbance at 470 nm was measured with a spectrophotometer.

### RNA isolation, transcriptome library construction, and RNA-seq data processing

The third to fifth leaves and 3–4 cm of the root tips of poplar 84 K were harvested, immediately frozen in liquid nitrogen, and stored at −80°C until use. Total RNA was extracted with an RNeasy Kit (Qiagen) according to the manufacturer's instructions. After purification and DNA digestion with RNase-free DNase (Qiagen), RNA quality was determined with a NanoDrop ND-2000 (A_260_/A_280_ 1.9–2.1), and an Agilent 2100 Bioanalyzer (28S/18S 1.8–2.0). RNA (3 µg per sample) was used to construct a strand-specific RNA-seq library on the Illumina HiSeq 4000 platform according to the manufacturer's instructions and index codes. Library construction and paired-end sequencing were performed by Beijing Novogene Technologies. Briefly, clean reads acquired after quality control and elimination of adapter- and poly(N)-containing reads as well as low-quality reads were mapped onto the reference genome of poplar 84 K (https://doi.org/10.6084/m9.figshare.12369209) as previously described (Huang et al., 2021) with TopHat (v. 2.0.0) with default parameters (Trapnell et al., 2014). Transcript levels were normalized based on fragments per kilobase of transcript per million fragments (FPKM) with Cufflinks (v. 2.1.1) with default options (Trapnell et al., 2014). Genes with >2-fold change and *P* < 0.05 (adjusted for the false discovery rate, *Q* < 0.05) were considered significantly differentially expressed.

### RT-qPCR of DEGs

To evaluate the expression of DEGs, we extracted total RNA with a Plant Qiagen RNeasy Kit (Qiagen China, Shanghai) following the manufacturer's instructions and purified it using the RNase-Free DNase Set (Qiagen). mRNAs were reverse-transcribed into cDNA with the Reverse Transcription System (Promega, Madison, WI, USA) according to the manufacturer's instructions. Gene-specific primers were used for RT-qPCR on a 7500 Fast Real-Time PCR System with SYBR Premix Ex Taq (TaKaRa). Reactions were performed with three technical and biological replicates with poplar actin (accession number: EF145577) as the internal control. The PCR program was as follows: initial denaturation at 94°C for 5 min; 40 cycles of 94°C for 30 s, 58°C for 30 s, and 72°C for 30 s; and a final melting curve from 70°C to 95°C. The sequences of primers are listed in Supplemental Table 1.

### GO enrichment analysis

DEGs were subjected to GO analyses with the AmiGO Term Enrichment tool (http://amigo.geneontology.org/). The R module CLUSTERPROFILER available at Bioconductor (http://bioconductor.org) was used to identify enriched GO terms associated with DEGs via hypergeometric probability. We applied multiple testing corrections using the Benjamini and Hochberg (1995) FDR method (Green and Diggle, 2007). GO terms with a corrected *P* < 0.05 were considered significantly enriched.

### Bioinformatics and phylogenetic analyses

The isoelectric point (pI) and molecular weight of GRF were predicted with ExPASy (http://web.expasy.org/compute_pi/). GRF sequences were subjected to multiple sequence alignment in MEGA X (Kumar et al., 2018). To address gaps and missing data, we selected partial deletion with a site coverage cutoff of 80%. The optimal amino acid substitution model was identified as Jones–Taylor–Thornton + (G) + (F). A phylogenetic tree of protein sequences was constructed with the maximum likelihood (ML) approach with 1,000 bootstrap replicates in MEGA X. All positions with <90% site coverage were eliminated; in other words, < 10% total alignment gaps, missing data, and ambiguous bases were allowed for any position. Figtree (http://tree.bio.ed.ac.uk/software/figtree/) was used to visualize the phylogenetic tree.

### Construction of a multi-layered hierarchical gene regulatory network using the backward elimination random forest algorithm

A total of 274 candidate ion transporter genes were evaluated using PlantPAN v.2.0 to identify the cis-regulatory elements in their 2-kb promoter regions (Chow et al., 2016). Based on 80% confidence values, these motifs were predicted to be target sites of 28 TFs. Then, the metal ion transporter, photosynthetic and oxidoreductase activity pathway genes, and TFs were used to construct a multi-layered hierarchical gene regulatory network (ML-hGRN) by employing the backward elimination random forest algorithm (Deng et al., 2017).

### Measurement of tissue specificity

Tissue-specific sampling included samples of leaf, root, apex, mature xylem, immature xylem, cambium, phloem, bark, and petiole. All samples were taken from the 1-year-old *Populus tomentosa* clone “LM50” planted in Guanxian County and promptly placed into liquid nitrogen. All transcriptome data of tissue specificity analysis used in this study are provided in Supplemental Table 9. All transcriptome data have been uploaded to the public database. The transcriptome expression data (three biological replicates per group) are available in the National Center for Biotechnology Information SRA database under accession numbers PRJNA521855, PRJNA521819, PRJNA522886, PRJNA357670, and PRJNA522891. The FPKM (fragments per kilo base of transcript per million fragments) method was used to normalize transcript expression. The processing of transcriptome data was described in a previous study (Quan et al., 2019).

### RT-qPCR analysis of mRNAs

A 7500 Fast Real-time PCR System (Applied Biosystems) was used for RT-qPCR analyses with SYBR Premix Ex Taq (TaKaRa). The cDNA template for reactions was reverse-transcribed using the total RNA extracted from leaves subjected to the salt stress treatments. The real-time data generated were analyzed using the Opticon Monitor Analysis Software 3.1 tool (Bio-Rad), and quantities of mRNA were standardized to the transcript levels of poplar ACTINII-like (accession number EF145577) and 18S ribosomal RNA (18S rRNA), which were used as internal controls, and then calculated using the 2^−ΔΔCT^ method (Livak and Schmittgen, 2001). The PCR program was as described by Zhang et al. For all reactions, three technical and biological repetitions were performed, and the specificity of the amplified fragments was checked using generated melting curves.

## Association analysis

Total genomic DNA (gDNA) was isolated from fresh leaves of 435 unrelated individuals from the *P*. *tomentosa* association population with DNeasy Plant Mini Kits (Qiagen). SNP calling was described previously (Xiao et al., 2020). Briefly, high-quality SNPs with uniquely mapped paired-end reads were selected to perform SNP calling using SOAPsnp with default parameters and VCFtools (Danecek et al., 2011). VCFtools was used to extract the gene-derived bi-allelic SNPs. snpEff was used to annotate SNPs, including synonymous and non-synonymous SNPs in the exon region. Single SNP-trait association analyses (MAF > 5%, missing < 20%) were performed by combining 29 traits (classified into six categories) in the 435 individuals (Zhao et al., 2021) with the mixed linear model (MLM) of TASSEL (v. 5.0), and phenotypes were normalized based on the Z score. The MLM considers the effects of population structure (Q) and kinship coefficients (K). The Q and K matrix was obtained as described by Du et al. (2019). QVALUE in R was used to correct for multiple testing using the positive FDR method (Storey, 2003). The significance level for single SNP-based association results was *P* < 0.001 and *Q* < 0.05.

### Multi-SNP epistatic interaction analysis

The EPISNP1 package integrated in the epiSNP_v.4.2 software was used to detect single-locus effects and two-locus epistasis interactions, based on the extended Kempthorne model. Single-locus effects including SNP genotypic effect, additive, and dominance effects, and epistatic effects including two-locus epistatic interaction (I), additive × additive (A × A), additive × dominance (A × D), dominance × additive (D × A), and dominance × dominance (D × D) were generated. Multifactor Dimensionality Reduction 3.02 (MDR3.0.2) was applied to investigate the genotype combinations and effects in our study. All *P*-values were corrected using Benjamini–Hochberg methods, and the significance level was set at *P* < 0.001.

### Identification of potential miRNAs regulating *GRFs* in poplar

Regulatory mechanisms and potential target sites of miRNAs were predicted with PsRNATarget (http://plantgrn.noble.org/psRNATarget/) based on 401 miRNA sequences of *Populus trichocarpa* (Puzey et al., 2012). Parameters were set as follows: maximum expectation score, 3; penalty for other mismatches, 1.0 (Dai and Zhao 2011). Degradome sequencing of a pool of six tissues from *P*. *tomentosa* (leaf, shoot apex, phloem, cambium, developing xylem, and mature xylem) was used to verify the prediction results. The data set is publicly accessible at http://bigd.big.ac.cn/gsa under the accession number CRA000989 (Quan et al., 2019).

### Plasmid construction and plant genetic transformation

To generate overexpression lines, we subjected poplar 84 K plants to cloning, expression analysis, and genetic transformation. Cuttings were grown under long-day conditions (16-h light/8-h dark). Transgenic 84 K poplars expressing the β-glucuronidase gene under the control of *PagHAK6* overexpression driven by the CaMV 35S promoter were generated based on previous study (He et al., 2018). We amplified the coding sequence (CDS) of *PagHAK6* from 84 K cDNA to construct the *PagHAK6* overexpression vector. Next, the sequence was cloned into the pBI121 vector and verified by sequencing. The constructed vector was introduced into *Agrobacterium tumefaciens* strain GV3101 and transformed into poplar 84 K by the leaf-disc method. Leaf discs were used for *Agrobacterium*-mediated transformation as described by Horsch et al. (1986): Discs were incubated for 10 min with *Agrobacterium* (OD_600_ 0.4–0.8) harboring the *35S::mPagHAK6* vector and incubated in darkness for 3 days on shoot-induction medium (Murashige-Skoog [MS] basal medium containing 0.5 mg L^−1^ 6-benzylaminopurine [6-BA] and 0.05 mg L^−1^ naphthaleneacetic acid). Transgenic plants were confirmed by PCR with vector- and gene-specific primers.

## 3,3-Diaminobenzidine (DAB) staining of transgenic plants

DAB staining was used to detect H_2_O_2_ as described previously (Ding et al., 2015a, 2015b). Briefly, leaves of 3-month-old transgenic and WT plants were treated with 200 mmol NaCl for 0, 1, and 3 h and infiltrated in 0.1 mg mL^−1^ DAB solution in darkness for 3 h at 28°C. To remove chlorophyl, we incubated stained leaves in 75% ethanol and photographed them.

### Measurement of net Na^+^ flux

Net flux of Na^+^ was measured with NMT (Younger USA, Amherst, MA, USA) and imFluxes (Younger USA, Amherst, MA, USA). For Na^+^ flux, 3-month-old individuals were treated with 100 mM NaCl for 24 h and incubated in measurement solution (Na^+^: 0.1 mM CaCl_2_, 0.1 mM KCl, 0.5 mM NaCl, and 0.3 mM MES, adjusted pH to 6.0 with Tris–HCl [pH 8.8]) for 5 min. The microsensor was calibrated with 0.5 or 5 mM NaCl in a calibration solution (0.1 mM CaCl_2_, 0.1 mM KCl, 0.3 mM MES [pH 6.0]) for the measurement of Na^+^ flux and to pH 5.5 or 6.5 in calibration solution (0.1 mM CaCl_2_, 0.1 mM KCl, and 0.3 mM MES) for the measurement of H^+^ flux. Ion fluxes were measured about 300 µm from the root tip, and data were exported with JCal (v. 3.3; a free MS Excel spreadsheet, http://www.youngerusa.com or http://www.xbi.org). Consumables were provided by Xuyue (Beijing) Sci. & Tech (Beijing, China; Sa et al., 2019).

### Y2H and Y2H-seq assays

Y2H assays were conducted to verify the physical interactions between proteins and TFs. The *PagGRF1*5 CDS was cloned into pGBKT7 (BD; Clontech), and interacting protein genes were inserted into pGADT7 (AD; Clontech). We transferred recombinant plasmids into yeast using a Y2H Kit (Weidi Biotechnology, Shanghai, China). Transformants were plated on SD–Leu–Trp medium and incubated for 2 days at 30°C. Interactions were tested on SD–Trp–Leu–His–Ade medium for 3–4 days at 30°C. For Y2H-seq, the cDNA libraries were then commercially constructed by OEbiotech (Shanghai, China). mRNAs were extracted from 2-month-old 84 K cuttings and reverse-transcribed to cDNA. Next, cDNA was inserted into the pDONR222 and pGADT7-DEST vectors. We transferred recombinant plasmids into the yeast strain using Y2HGold Kit (Weidi Biotechnology Co. Ltd, Shanghai, China). All transformants were placed on SD–Leu–Trp plates and incubated for 2d at 30°C. Interactions were tested on SD–Trp–Leu–His–Ade medium for 3–4 days at 30°C. We used pGBKT7-*PagGRF15* plasmids to verify self-activation and screen *PagGRF15*-interacting proteins using the Y2H mating protocol (OEbiotech). We selected and mixed 10 events in one PCR tube with 50 µL ddH_2_O. Next, we added 1-µL liquid to each tube as the PCR template. To maximize the yield of DNA fragments of *PagGRF15*-interacting proteins, we increased the extension time to 3 min if extension efficiency was <1 kb/min. To extract as many DNA fragments of PagGRF15-interacting proteins as possible, the PCR program was carried out with an initial denaturation at 95°C for 5 min, which was followed by 50 cycles of 94°C for 30 s, 56°C for 30 s, and 72°C for 30 s. PCR products were mixed in a 10-mL tube for purification using the agarose gel electrophoresis method (Niu et al., 2022) prior to being sequenced using the NGS method by Beijing Novogene Technologies. Finally, Cytoscape (Smoot et al., 2011) was used for displaying all modules.

## DAP-seq and data processing

DAP-seq was performed as described previously (Bartlett et al., 2017). The *PagGRF15* CDS was cloned into the pFN19K vector. PagGRF15 was expressed with a TnT® Coupled Wheat Germ Extract System (Promega). gDNA was extracted from leaves harvested from 3-month-old 84 K poplar with a DNeasy Plant Mini Kit (Qiagen, Germany). gDNA has sonicated to a fragment size of 200–800 bp. Halo-*PagGRF15* protein was bound to anti-Halo monoclonal antibody agarose beads (Promega) and incubated with 200-ng fragmented gDNA for 1 h at room temperature. The beads were washed, and DNA was recovered. Samples were pooled and sequenced on an Illumina Novaseq 6000 platform; 150-bp paired-end reads were generated from each library. A total of 10–30 million reads were obtained for each sample.

Sequencing reads were trimmed with Trimmomatic with the following parameters: ILLUMINACLIP: TruSeq3-PE.fa:2:30:10:8:true, LEADING: 20, TRAILING: 20, SLIDINGWINDOW: 4:20, MINLEN: 50. Trimmed reads were mapped to the poplar 84 K genome (https://doi.org/10.6084/m9.figshare.12369209) (Huang et al., 2021) with Bowtie2 (v. 2.3.5). Using SAMtools, we filtered mapped reads to obtain uniquely mapping reads, which were used in subsequent analyses. Peak calling was done with MACS2. Associations of DAP-seq peaks 2 kb upstream or downstream of the transcription start site were analyzed with BEDtools according to General Feature Format files. Visualization of peak coverage over chromosomes and profiles of peaks binding to TSS regions was performed with ChIPseeker (v. 1.22.1). Motif discovery was conducted with MEME-ChIP suite (v. 5.0.5; Machanick and Bailey, 2011).

### Yeast one-hybrid assay

Y1H assays were performed to verify physical interactions between promoters and TFs. The 220-bp promoter fragment of *PagHAK6* was amplified from poplar 84 K and cloned into the pAbAi vector with the primers in Supplemental Table 1. The CDS of *PagGRF15* and *PagERF1* was inserted into the pGADT7 vector to generate recombinant GAD-PagGRF15 and GAD-PagERF1, respectively. Y1H assay was performed with the Matchmaker Gold Y1H Library Screening System according to the manufacturer's instructions (Clontech Laboratories, Mountain View, CA, USA). pGAD-Rec2-53 and pAbAi-promoter, pGAD-PagGRF15/pGAD-PagERF1 and pAbAi, and pGAD-Rec2-53 and pAbAi were used as negative controls. pGAD-Rec2-53 and p53pAbAi (provided in the kit) were used as positive controls. The plasmids were co-transformed into Y187 yeast, which was plated on SD–Trp/–Leu/–His medium containing 200–800 ng mL^−1^ Aureobasidin A (AbA) as described previously (An et al., 2018).

### Dual-luciferase reporter assay

The full-length ORF of *PagGRF15* and *PagERF1* was cloned into the pGreenII 62-SK effector vector, and the promoter of *PagHAK6* (220 bp) used in the Y1H assay was cloned into the pGreenII 0800-LUC reporter vector. The recombinant plasmids and negative control vectors were introduced into *A*. *tumefaciens* GV3101 (pSoup-p19). The effector and reporter vectors were co-transformed into *Nicotiana benthamiana* leaves, as previously described (Yao et al., 2020). D-Luciferin (10 µM) was sprayed onto *Nicotiana benthamiana l*eaves and then photographed using an LB985 NightSHADE fluorescence imaging system (Berthold Technologies, Bad Wildbad, Germany). Dual-luciferase (LUC) activity was determined using a GloMax 20/20 luminometer (Promega, Madison, WI) and a Dual-Luciferase Assay Kit (Promega) according to the manufacturer's instructions. The experiments were performed at least three times with six technical repeats.

### BIFC assays

The CDS of *PagGRF15* was inserted into the 35S-pspyCe-YFP vector. The CDS of *PagERF1* was inserted into the 35S-pspyNe-YFP vector. The primers are shown in Supplemental Table 1. The obtained recombinant plasmids 35S:PagGRF15-CYFP and 35S:PagERF1-NYFP were introduced into *A. tumefaciens* GV3101 competent cells and further co-transferred into *Allium cepa* endoepidermal cells for 24- to 48-h culture. Finally, the fluorescence of YFP was detected using the confocal laser scanning microscope (Olympus BX53F, Tokyo, Japan), the complete experimental setup (i.e. lasers, intensity, collection bandwidth, and gains) used for the confocal work based on the previous study (He et al., 2018).

### Subcellular localization of PagGRF15 and PagHAK6

The CDS of GRF15 and HAK6 was inserted into the pHB vector, and the recombinant vector plasmid was introduced into *A. tumefaciens* strain GV3101. GV3101 was redissolved in the infiltration buffer (10 mM MgCl_2_, 10 mM MES, and 150 µM acetosyringone). Then, the mixed bacterial solution was injected into 28-day-old tobacco (*Nicotiana tabacum*) leaves. The confocal laser microscope LSM880 (Carl Zeiss, Oberkochen, Germany) was used to image the signal after 2-d injection, the complete experimental setup was based on a previous study (Zhang et al., 2019). The primers are shown in Supplemental Table 1.

### Statistical analysis

Microsoft Excel 2010 (Microsoft, Redmond, WA, USA) and SPSS (v. 17.0; IBM, Chicago, IL, USA) was used for data analysis. One- and two-way analyses of variance were used to determine the significance of differences among treatments. Student's *t*-test was used to calculate *P*-values (*P < 0.05, **P < 0.01). The data were normalized, and all samples were normally distributed in terms of homogeneity of variance.

### Accession numbers

Gene sequence data were available online with accession numbers Pop_A14G044741 (*PagGRF15*) and Pop_G19G021904 (*PagHAK6*). Raw data for RNA-seq, DNA affinity purification sequencing (DAP-seq), and small yeast two-hybrid-sequencing (Y2H-seq) are available at the BIGD Genome Sequence Archive (https://bigd.big.ac.cn) under accession number CRA006695.

## Supplementary Material

kiac600_Supplementary_Data

## Supplemental data

The following materials are available in the online version of this article.

**Supplemental Table S1**. Primers used in this study.

**Supplemental Table S2**. Amino acid sequences, pIs, and molecular masses of plant GRF genes in this study.

**Supplemental Table S3**. GO enrichment analysis of all DEGs under salt stress.

**Supplemental Table S4**. GO enrichment analysis of DEGs under salt stress in leaf.

**Supplemental Table S5**. GO enrichment analysis of DEGs under salt stress in root.

**Supplemental Table S6**. Genes differentially expressed in poplar 84 K in response to salt stress.

**Supplemental Table S7**. TFs differentially expressed in poplar 84 K in response to salt stress.

**Supplemental Table S8**. Promoter enrichment in clusters L1 to R4 in this study.

**Supplemental Table S9**. All transcriptome data of tissue specificity analysis used in this study.

**Supplemental Table S10**. Interacting proteins detected by Y2H-seq.

**Supplemental Table S11**. Target genes of PagGRF15 detected by DAP-seq.

**Supplemental Table S12**. GO enrichment analysis of target genes of PagGRF15.

**Supplemental Table S13**. Interacting proteins correlated with PagGRF15.

**Supplemental Table S14**. Amino acid sequences, pIs, and molecular masses of plant HAK genes.

**Supplemental Table S15**. Potential regulation of PagGRFs by miRNAs.

**Supplemental Table S16**. Significant SNPs associated in TFs and PTGs with traits in the association population of *P*. *tomentosa*.

**Supplemental Table S17**. Expression pattern of differentially expressed genes in two tissues under salt stress.

**Supplemental Figure S1**. A three-layered GRN mediated by PagGRF15.

**Supplemental Figure S2**. Poplar phenotype under salt stress is significantly affected by allelic loci within candidate genes across the three-layer gene regulatory network.

**Supplemental Figure S3**. PagGRF15 positively regulates phenotypic traits in poplar 84 K.

**Supplemental Figure S4**. PagGRF15 positively interacts with protein in poplar 84 K.

**Supplemental Figure S5**. The interaction between GRF15 and ERF1.

**Supplemental Figure S6**. PagGRF15 positively regulates HAK genes.

**Supplemental Figure S7**. Hot spot map and distribution frequency of reads at 2.5 K upstream and downstream of TSS.

**Supplemental Figure S8**. Enhanced salt tolerance in transgenic poplar overexpressing PagHAK6.

**Supplemental Figure S9**. Bioinformatics analysis of HAK gene.

**Supplemental Figure S10**. The potential regulation of PagHAK6 gene.

**Supplemental Figure S11**. PagERF1 positively regulates PagHAK6.

**Supplemental Figure S12**. Model of the regulatory network of K+/Na + homeostasis and ROS scavenging involving PagGRF15, PagHAK6, and PagERF1 in Populus under salt stress.
