# Supplementary material for: Growth-regulating factor 15-mediated gene regulatory network enhances salt tolerance in poplar
Source: Plant Physiol. 2022 Dec 26;191(4):2367–84. doi: 10.1093/plphys/kiac600 (PMC10069893; doi:10.1093/plphys/kiac600)
Supplement: kiac600_Supplementary_Data [file kiac600_supplementary_data.zip › Supplemental Figures S1S12.pdf]

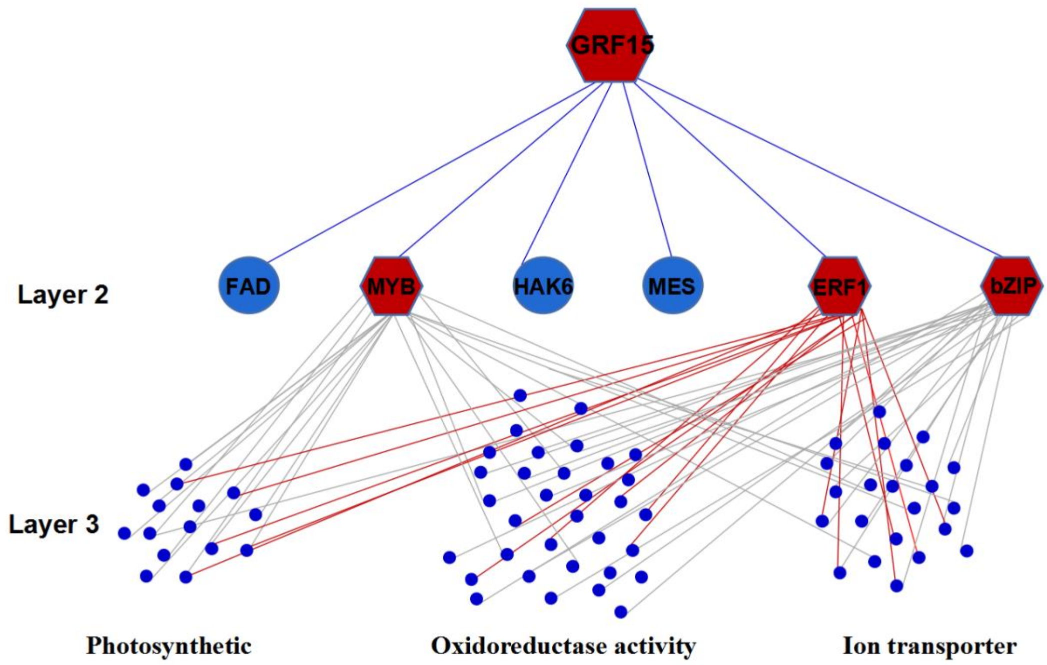


**Supplemental Figure S1.** A three-layered GRN mediated by *PagGRF15*. Each line represents a regulatory relationship inferred by top-down GGM algorithm and motif analysis. TFs as hexagons, and genes as circles. The thickness of each connecting line reflects the relative strength of the proposed interaction. Red lines represent the connection of *PagERF1* and its downstream targets, blue lines represent *PagGRF15* and its targets, gray lines represent the connection of other TFs and its downstream targets. TF mean transcription factor.

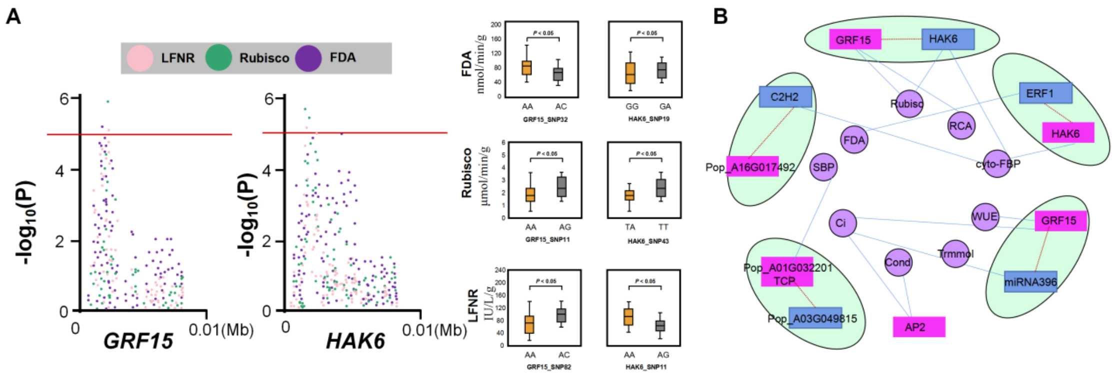


**Supplemental Figure S2. Poplar phenotype under salt stress is significantly affected by allelic loci within candidate genes across the three-layer gene regulatory network.** Manhattan (A) and quantile-quantile plots from marker-trait association analysis between DE-TFs or DE-PTGs and phenotypic traits in the association population. (B) Epistatic effects of salt-responsive genes (DE-TFs and their DE-PTGs) for 25 phenotypic traits. LFNR mean leaf type ferredoxin NADP$^+$ oxidoreductase, Rubisco mean Ribulose bisphosphate carboxylase oxygenase and FDA mean fructose-1,6-bisphosphate aldolase. Magenta and blue rectangles represent responsive genes and TFs, respectively, purple circles represent the phenotypic traits, red dotted lines represent TFs-target pairs. In boxplot, center line: the value in the middle after the data is sorted from small to large; box limits: Maximum and minimum of the value; upper (Q2) and lower (Q1) quartile mean number at 75% of the data series and number at 25% of the data series; 1.5x interquartile range: = Q3-Q1. Bottom edge and upper edge mean Q1 - 1.5 *IQR and Q3 + 1.5 *IQR respectively.

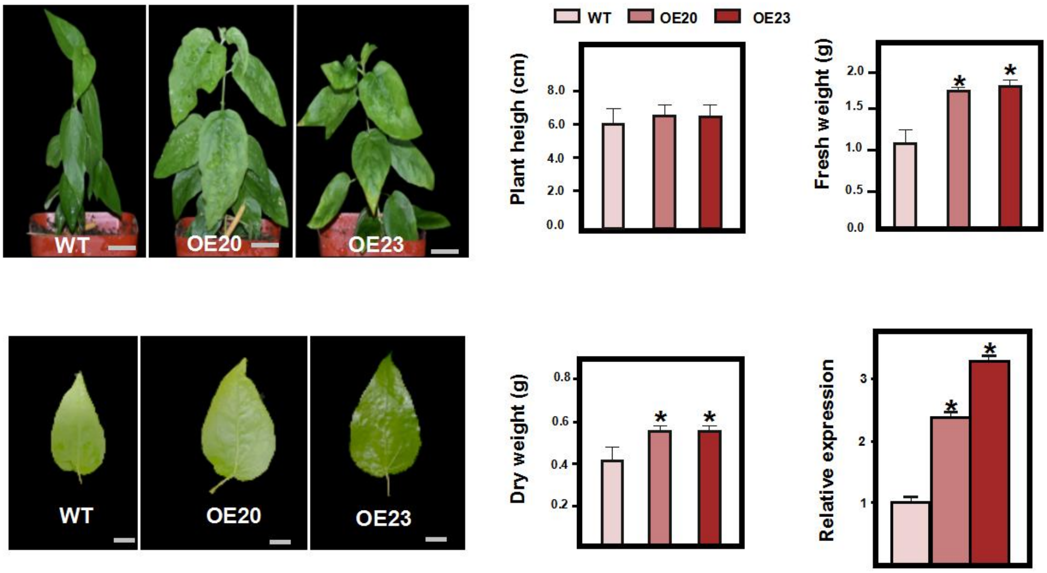


**Supplemental Figure S3.** ***PagGRF15*** **positively regulates phenotypic traits in poplar 84K** (A) The phenotypes of WT and transgenic (OE) plants under normal condition. The individual images in the bottom section were digitally extracted for comparison. Bars, 2 cm. (B–D) Plant height (PH), fresh weight (FW), and dry weight (DW) of WT and OX plants. Scale bars, 2 cm. (E) RT-qPCR of two PagGRF15-transformed poplar lines (OE). *PagActin* was used as a reference for normalization. *Significant difference between WT and OX plants (*$P < 0.05$). Values are means ± SE (n = 8).

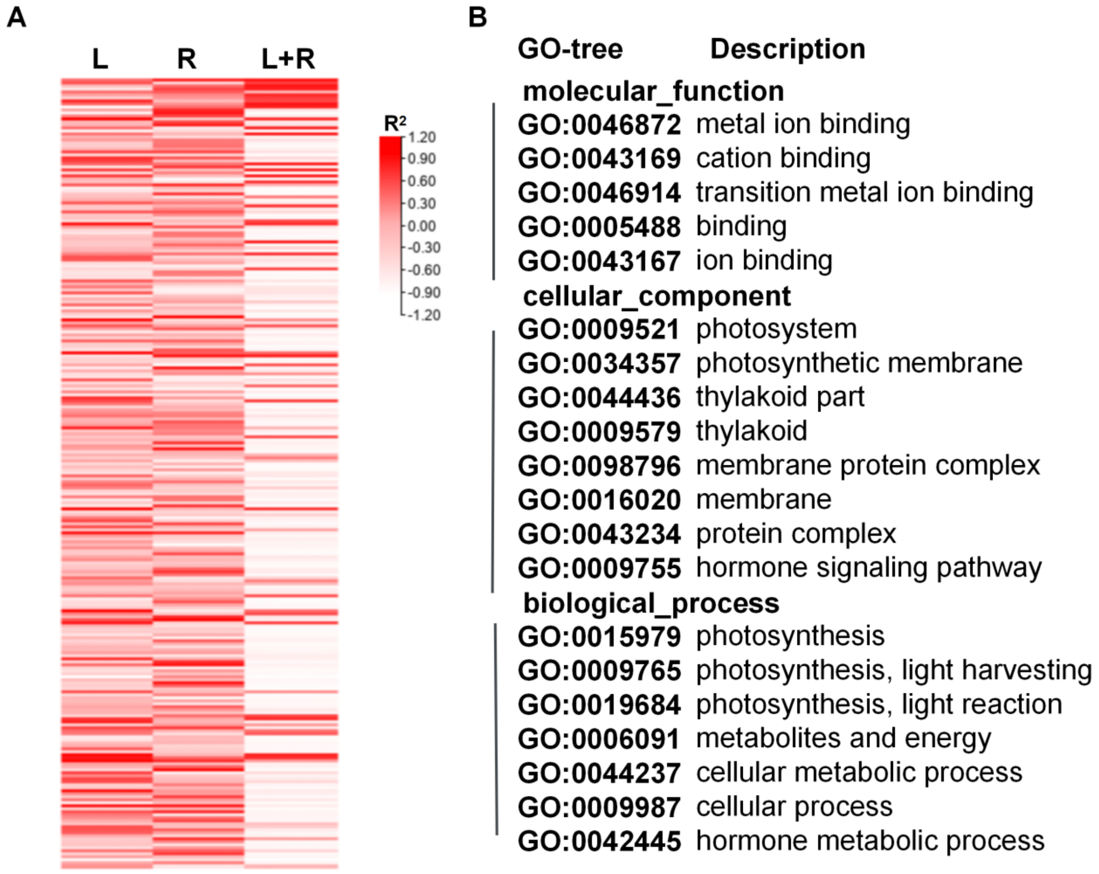


**Supplemental Figure S4. *PagGRF15* positively interacts with protein in poplar 84K** (A) Expression of PagGRF15-interacting protein genes under salt stress. The heat map shows the degree of similarity of expression in leaf and root. L, leaf; R, root; L+R, both leaf and root. (B) GO analysis of 82 PagGRF15-interacting protein genes with positive correlations.

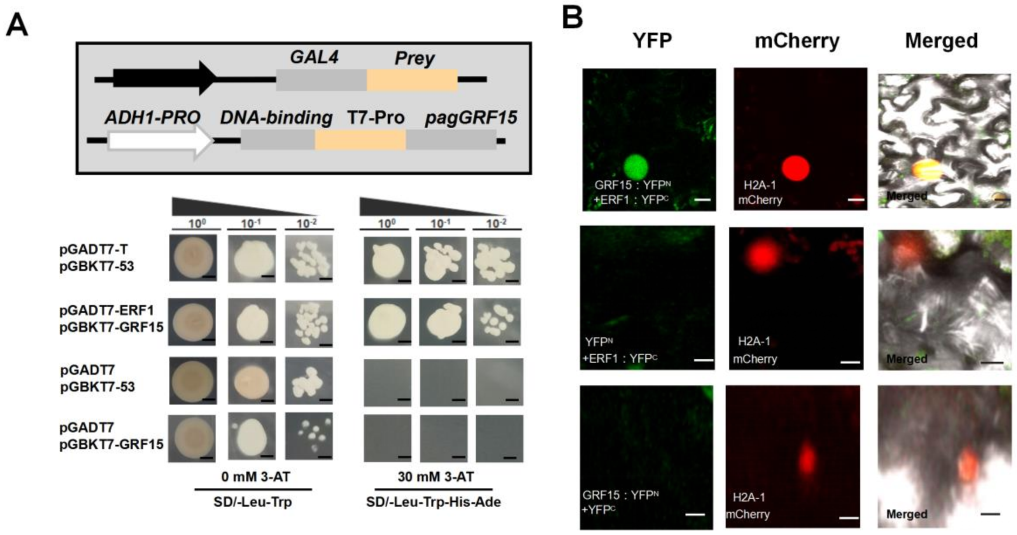


**Supplemental Figure S5. The interaction between GRF15 and ERF1**. (A) Y2H assay of interactions between PagGRF15 and PagERF1. pGADT7-T and pGBKT7-53 were used as positive controls; pGADT7 and pGBKT7-53, and pGADT7 and pGBKT7, were used as negative controls. Yeast dilution factors: $10^0$, $10^{-1}$, and $10^{-2}$ (left to right). Bars, 0.25 cm. (B) BIFC assay Interaction between PagGRF15 and PagERF1 in vivo. Neither negative control gave any YFP signal. Green shows the YFP signals from protein interaction, red indicates the nuclear marker H2A-1:mCherry, and yellow represents the merged signals from YFP and mCherry. Bars, 0.20 μm.

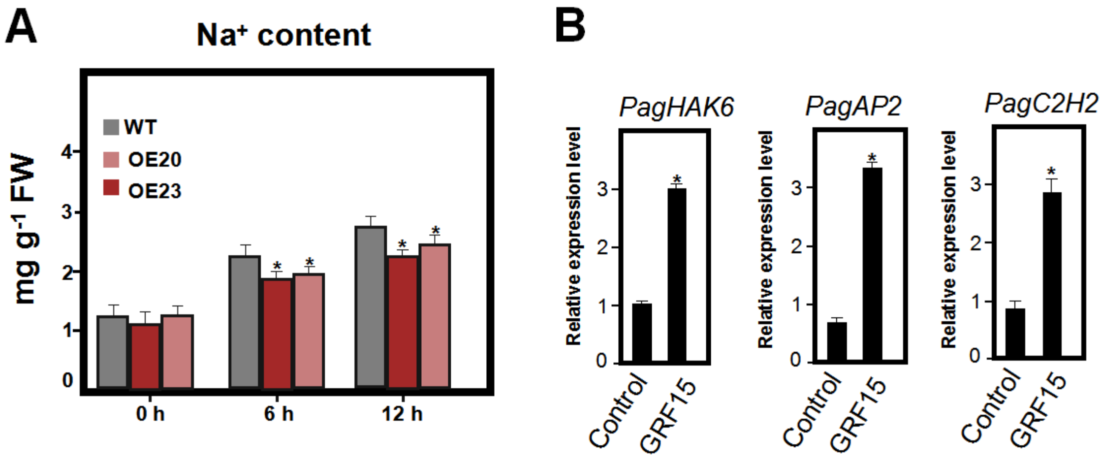


**Supplemental Figure S6. *PagGRF15* positively regulates *HAK* genes** (A) $Na^+$ content of cuttings of WT and 35S:PagGRF15 plants under normal and salt stress conditions. (B) RT-qPCR to detect the transcript abundance of PagHAK6, PagAP2, and PagC2H2 in wild type (Control) and *PagGRF15* overexpressing cuttings (GRF15). Asterisks indicate significant differences for each gene between control and those overexpressing PagGRF15 samples for each gene (*, $P < 0.01$, Student's t-test). Values are means ± SE (n = 20).

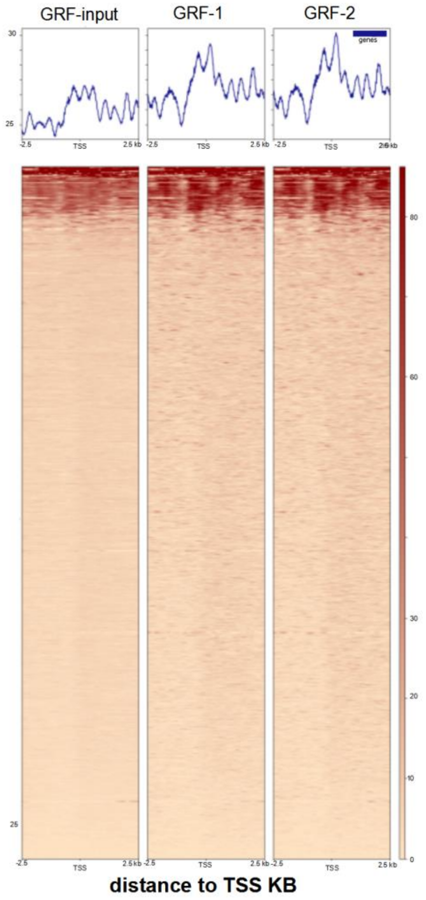


**Supplemental Figure S7.** Hot spot map and distribution frequency of reads at 2.5 K upstream and downstream of TSS. TSS: Transcription Start Sites.

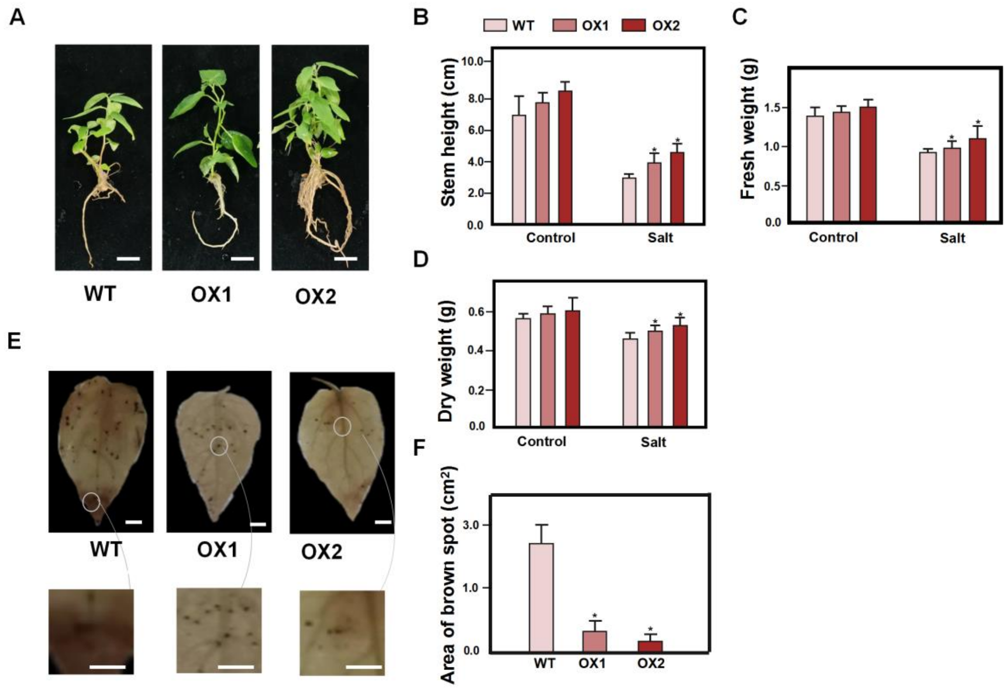


**Supplemental Figure S8. Enhanced salt tolerance in transgenic poplar overexpressing *PagHAK6*.** (A) Shoot and root phenotypes of WT and transgenic (OX) plants. Scale bars, 2 cm. (B–D) Plant stem height (SH), fresh weight (FW), and dry weight (DW) of WT and OX plants. Cutting-propagated WT and OX plants were cultivated on solid 1/2 MS medium supplemented with 75 mmol NaCl for 15 days. SH, FW, and DW of individual plants. *Significant difference between WT and OX plants (*$P$ < 0.05, Student's t-test). (E) DAB staining of salt stress–induced ROS in leaves of WT and OX-PagHAK6 (OX1/2). Scale bars of upper, 1 cm. (F) Area of brown spotting per cross-sectional area ($cm^2$). *Significant difference between WT and OX plants (*, $P$ < 0.01, Student's t-test).

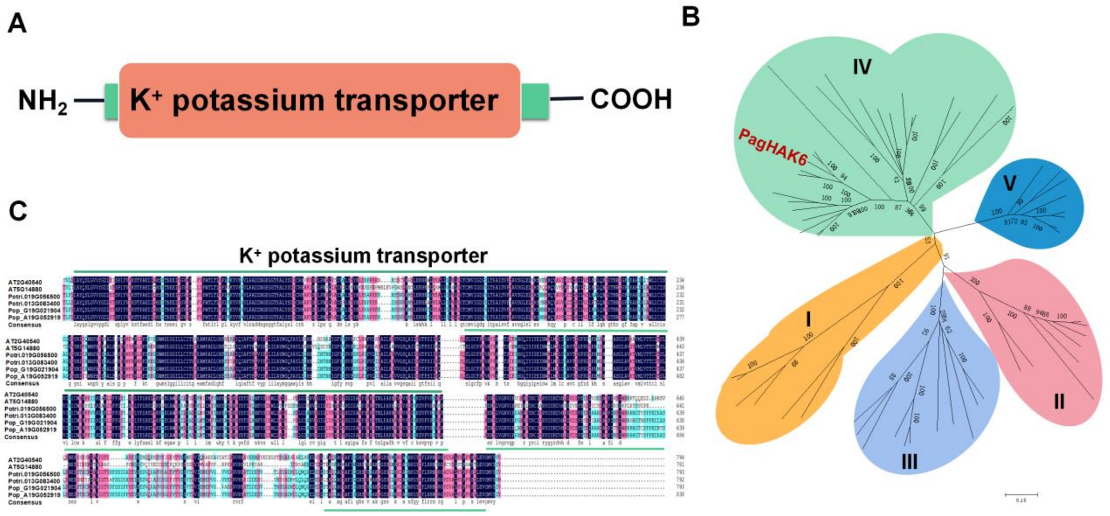


**Supplemental Figure S9. Bioinformatics analysis of *HAK* gene** (A) Domain architecture analysis of PagHAK. (B) Phylogenetic analysis of HAK homologs from *O. sativa*, *P. alba* × *P. glandulosa* 84K and *A. thaliana.* Scale bars, 0.1. (C) Multiple amino acid sequence alignment of PagHAK from poplar 84K (*P. alba* × *P. glandulosa*) and *A. thaliana*.

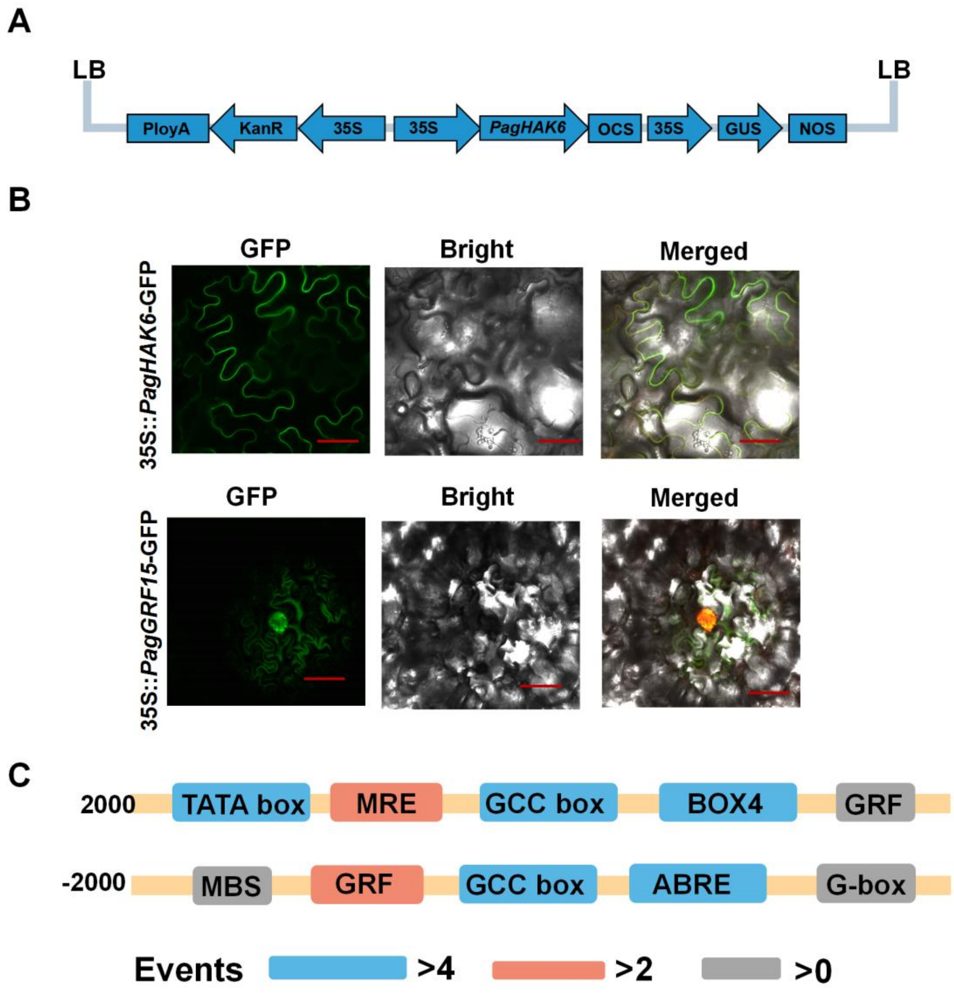


**Supplemental Figure S10. The potential regulation of *PagHAK6* gene** (A) 35S overexpression carrier of *PagHAK6*. (B) Subcellular localization of PagGRF15 and PagHAK6. Scale bars 80 μm. (C) Cis-acting elements of *PagHAK6*. The *PagHAK6* promoter was predicted with the Plant CARE database (http://bioinformatics.psb.ugent.be/webtools/plantcare/html/).

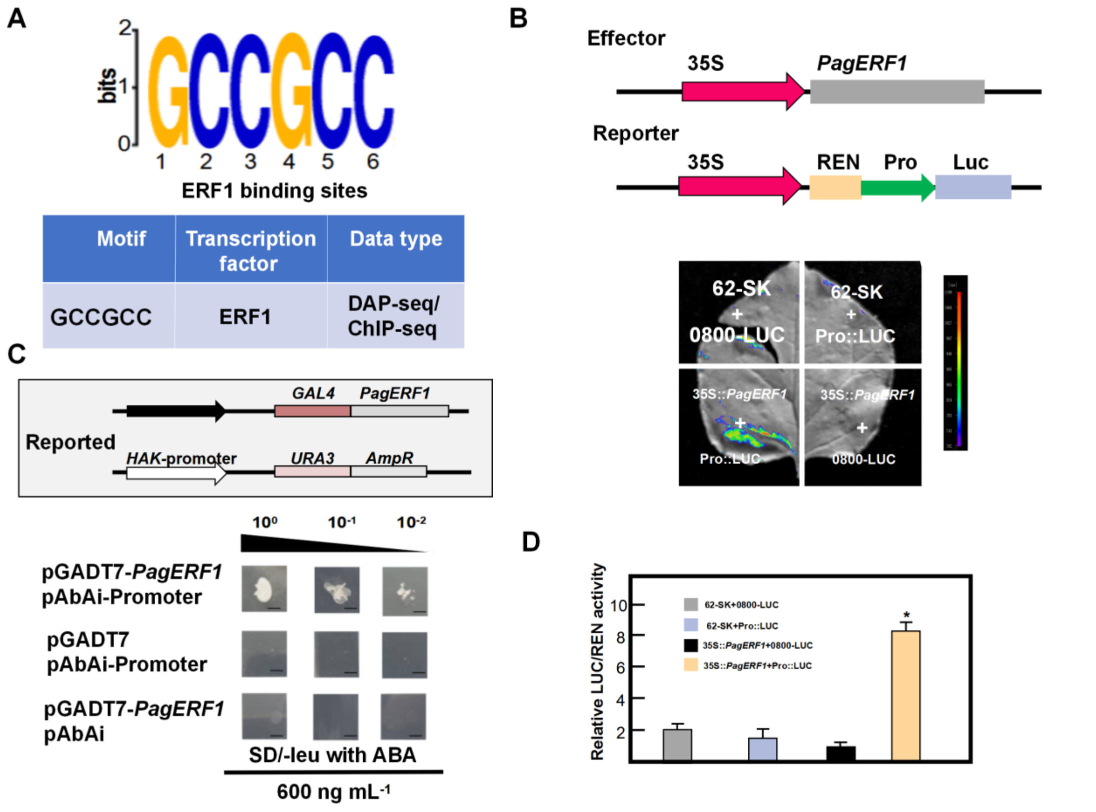


**Supplemental Figure S11. *PagERF1* positively regulates *PagHAK6*** (A) Bioinformatic analysis of PagERF1 binding site (TFBS) Motifs. (B) Dual-luciferase reporter analysis of *PagERF1* activating the *PagHAK6* promoter in *N. benthamiana* leaves. Schematic of the reporter and effector constructs is shown at the top. The effector and reporter vectors were instantaneously co-transformed into leaves of *N. benthamiana* and cultured in a greenhouse under control conditions for 48 h (Light culture for 24 hours, dark culture for 24 hours). The experiments were performed three times and the similar results were obtained. The bars represent means ± SD (n = 6) and the asterisks indicate significant differences (**P* < 0.05, Student' s t test). (C) Y1H analyses of the interaction between PagERF1 and the PagHAK6 promoter. Top, reporter and effector vectors. Reporter and effector constructs were co-transformed into yeast Y187 cells, and positive transformants were identified by spotting serial dilutions onto SD/-Leu medium supplemented with 800 ng $mL^{-1}$ AbA. pGADT7-53+pAbAi-Lam and pGADT7+pAbAi-promoter were constructed in the

same manner as the negative controls. Yeast dilution factors: $10^0$, $10^{-1}$, and $10^{-2}$ (left to right). Bars, 0.25cm.

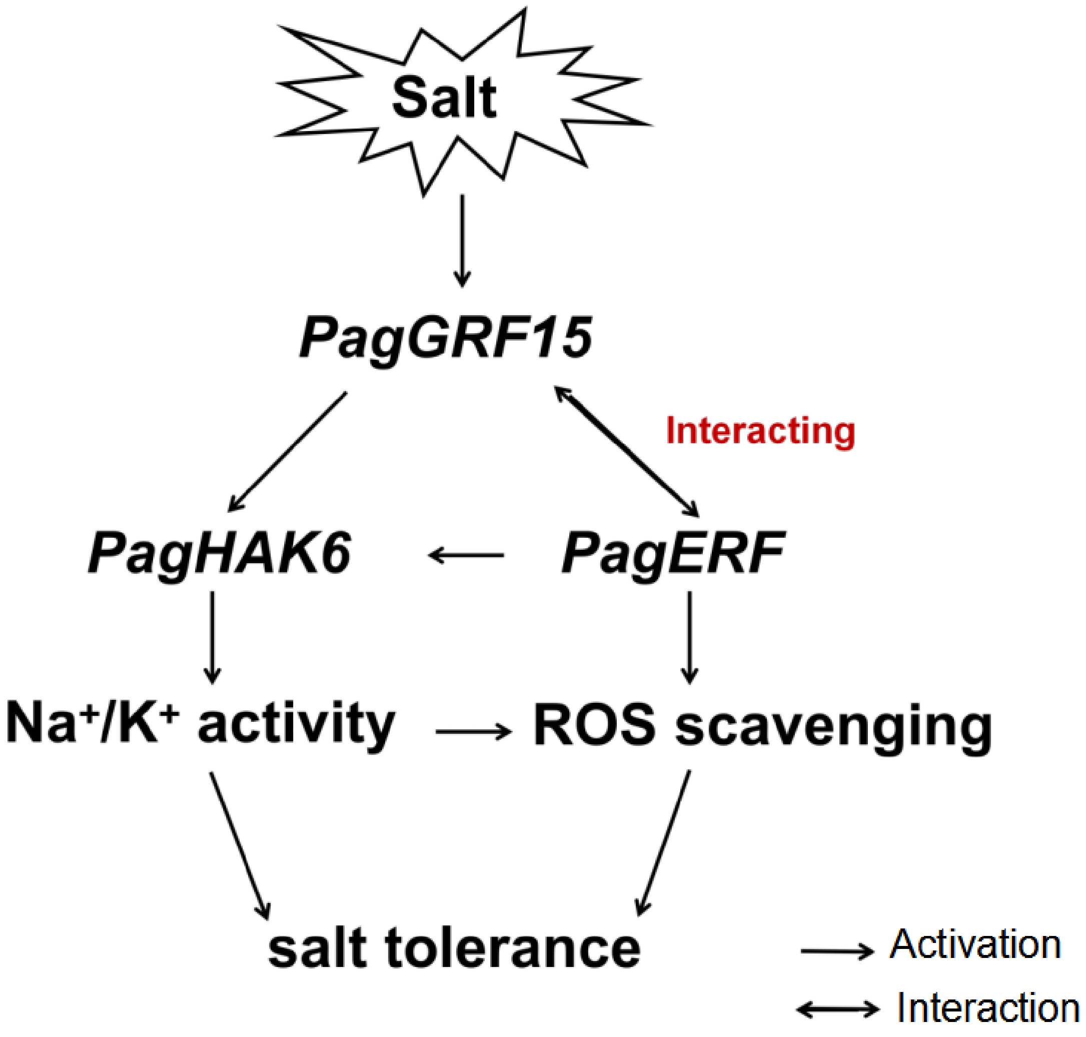


**Supplemental Figure S12. Model of the regulatory network of $K^+/Na^+$ homeostasis and ROS scavenging involving *PagGRF15*, *PagHAK6* and *PagERF1* in *Populus* under salt stress**. ROS mean reactive oxygen species.
